# Extracellular electron transfer proteins contribute to reduction of ferric minerals by *Geobacter* biofilms

**DOI:** 10.1128/aem.00369-25

**Published:** 2025-04-09

**Authors:** Jiacheng Xu, Wei Zhou, Xi Han, Jian Liu, Yiran Dong, Yongguang Jiang, Yuhong Zhong, Liang Shi, Yidan Hu

**Affiliations:** 1 School of Environmental Studies, China University of Geosciences 504988, Wuhan, Hubei, China; 2 State Key Laboratory of Geomicrobiology and Environmental Changes, China University of Geosciences 12564, Wuhan, Hubei, China; 3 Hubei Key Laboratory of Yangtze Catchment Environmental Aquatic Science, China University of Geosciences 12564, Wuhan, Hubei, China; 4 State Environmental Protection Key Laboratory of Source Apportionment and Control of Aquatic Pollution, Ministry of Ecology and Environment, China University of Geosciences 12564, Wuhan, Hubei, China; University of Nebraska-Lincoln, Lincoln, Nebraska, USA

**Keywords:** microbial iron reduction, extracellular electron transfer, ferric iron minerals, *Geobacter sulfurreducens *biofilm

## Abstract

To investigate how the thickness and extracellular electron transfer (EET) capabilities of microbial biofilms influence the reduction of ferric iron [Fe(III)]-containing minerals, we utilized four strains of *Geobacter sulfurreducens* with varying biofilm thicknesses and EET capabilities. These strains were engineered by modulating intracellular levels of dinucleotide second messengers. We systematically investigated the capacity of biofilms formed by four strains to reduce different Fe(III)-containing minerals including ferrihydrite, goethite, and lepidocrocite. By growing the *G. sulfurreducens* biofilm on the Fe(III) mineral-coated slides, our results showed that the strains forming thin biofilms on surfaces of Fe(III) minerals exhibited faster Fe(III) reduction rates compared to those with thick biofilms. Transcriptomic analyses revealed the upregulation of the genes encoding bacterial EET-involved proteins in the thin biofilms, highlighting the significant role of these proteins in reducing Fe(III)-containing minerals by *G. sulfurreducens* biofilms. Furthermore, genetic characterization identified the participation of two novel *c*-type cytochromes (*c*-Cyts), GSU1996 and GSU2513, in the reduction of Fe(III)-containing minerals by *G. sulfurreducens* biofilms. The results from this study provide an improved understanding of mineral–microbe interaction.

**IMPORTANCE:**

*Geobacter* is a predominant species within biofilm communities that facilitate iron reduction, a process essential for the biogeochemical cycling of iron and other elements. However, the specific properties of *Geobacter* biofilms crucial for iron reduction remain unclear. By manipulating intracellular levels of dinucleotide second messengers to generate strains with varying biofilm properties, this research reveals that thinner biofilms exhibit superior rates of ferric iron [Fe(III)] mineral reduction compared to thicker biofilms. This finding highlights the vital role of proteins involved in extracellular electron transfer (EET) in enhancing the reduction of Fe(III)-containing minerals. The study further identifies two novel *c*-type cytochromes, GSU1996 and GSU2513, as important contributors to this process. These discoveries not only advance our understanding of microbial iron reduction but also offer new perspectives on the interactions between biofilms and mineral surfaces, potentially informing future research and applications in biogeochemical cycling and bioenergy.

## INTRODUCTION

Ferric iron [Fe(III)]-oxyhydroxides are abundant in soils and sediments, and exist in variable mineral forms spanning a wide range of formal oxidation-reduction (redox) midpoint potential (1–4). In anoxic environments, dissimilatory metal-reducing microorganisms (DMRMs) thrive by coupling cytoplasmic respiratory oxidation reactions of electron donors to the reduction of extracellular Fe(III)-containing minerals (5, 6). Among DMRMs, *Geobacter* spp. have been extensively studied. They can grow via reduction of Fe (hydr)oxides with redox potentials ranging from +0.37 V [e.g., Fe(III) citrate] to –0.27 V (e.g., goethite, α-FeOOH) versus the standard hydrogen electrode (SHE) (7). As a result, *Geobacter* spp. commonly dominate in iron-rich habitats where they are key contributors to carbon and mineral cycling (8).

Bacterial cells frequently adhere to surfaces or to each other, producing a matrix of hydrated extracellular polymeric substances (EPS) to form biofilms (9). It is estimated that 40%–80% of bacterial and archaeal cells on Earth reside in biofilms to drive almost all biogeochemical processes (10). In natural settings, iron oxides often occur as reactive coatings on the surface of soils and sediment particles (11–13), providing abundant electron acceptors as well as substrates for microbial growth in a biofilm mode. *Geobacter* spp. have been identified as the major phylotypes in the attached fraction of samples from iron-rich environments (14, 15), highlighting their crucial role in biofilm-mediated Fe(III) reduction. In *Geobacter* biofilms, electrons are transferred from the quinone/quinol pool in the cytoplasmic membrane to the bacterial surface via multiheme *c*-type cytochromes (*c*-Cyts). These electrons are then further transferred to extracellular electron acceptors either directly through the outer membrane *c*-Cyts or via a conductive matrix (16, 17). Components in the EPS matrix, such as *c*-Cyts (18), pili (19), polysaccharides (20), and flagella (21), are crucial for the formation of *Geobacter* biofilms and influence extracellular electron transfer (EET). While considerable progress has been made in studying EET in *Geobacter* biofilms, much of this research has been conducted in electrochemical systems using electrodes as electron acceptors. Additionally, most studies on Fe(III) reduction by *Geobacter* have concentrated on planktonic cells (22–24), with relatively little focus on biofilm-mediated Fe(III) reduction in natural environmental conditions such as soil or sediment surfaces.

Biofilm formation is often regulated by sophisticated intracellular signaling networks that modulate the levels of small molecules (25). Cyclic di-GMP (c-di-GMP), a well-studied dinucleotide second messenger, has been shown to play a crucial role in governing both biofilm formation and EET in *Geobacter sulfurreducens* (26, 27). In our previous investigation, we constructed three *G. sulfurreducens* strains with varying intracellular c-di-GMP levels: PCA/GMP-L (low c-di-GMP), PCA/C (intermediate c-di-GMP), and PCA/GMP-H (high c-di-GMP) (26). These strains formed biofilms with varying thickness and exhibited different EET properties. Additionally, it has been reported that cyclic AMP-GMP (cGAMP), another dinucleotide second messenger, plays a crucial role in regulating the gene expression related to metal reduction in *G. sulfurreducens* (27). Furthermore, cGAMP promotes a transiently attached lifestyle that facilitates short and intermittent interactions between *G. sulfurreducens* and nanometer-sized particles. This lifestyle is distinct from the permanently attached biofilm lifestyle, which is signaled by c-di-GMP. Given the pivotal role of c-di-GMP and cGAMP signaling networks in regulating biofilm formation and EET in *Geobacter* species, targeted manipulation of these signaling pathways offers a promising strategy to modulate biofilm properties.

To investigate how the thickness and EET capabilities of microbial biofilms influence the reduction of Fe(III)-containing minerals, we modulated the thickness of *G. sulfurreducens* biofilms by altering intracellular levels of c-di-GMP and cGAMP. We systematically compared the Fe(III)-reducing abilities of biofilms formed by PCA/GMP-L, PCA/GMP-H, PCA/GAMP-H, and PCA/C on the surfaces of Fe(III)-containing minerals. Fe(III)-containing minerals ferrihydrite [Fe₂O₃•0.5H₂O], goethite [α-FeOOH], and lepidocrocite [γ-FeO(OH)] were selected due to their widespread occurrence in natural and engineered environments. Biofilm formation, Fe(III) reduction, and the resultant secondary iron minerals were analyzed. Additionally, RNA-seq-based transcriptomics and genetic investigations were performed to elucidate the role of EET proteins in biofilm-mediated Fe(III) reduction. This study provides valuable insights into the factors constraining the Fe(III) reduction capabilities of biofilms and advances our understanding of microbe–mineral interactions.

## MATERIALS AND METHODS

### Bacterial strains and culture conditions

The strains and plasmids used in this study are listed in Table S1 in the supplemental material. The wild-type (WT) strain *G. sulfurreducens* PCA, stocked in our lab, was purchased from the American Type Culture Collection (ATCC). The stability of the pYYDT vector (28) in *G. sulfurreducens* PCA was improved through a plasmid-host adaptation strategy (29). The copy number of the pYYDT vector was determined using previously reported methodologies (30). In brief, the detection of both plasmid and chromosomal DNA from *G. sulfurreducens* PCA was performed via quantitative PCR (qPCR) using two sets of primers specific for the *lacI* gene on the plasmid and for *omcS* on the chromosomal DNA, respectively. Given that both *lacI* and *omcS* are single-copy genes in pYYDT and chromosomal DNA, the plasmid copy number can be calculated as the ratio of *lacI* copies to *omcS* copies. The copy number of the pYYDT vector was found to be 48 ± 4 and 34 ± 1 before and after a lepidocrocite reduction experiment lasting 26 days without antibiotics, respectively, indicating the relative stability of the pYYDT vector in *G. sulfurreducens* PCA. The control strain PCA/C and the engineered strains PCA/GMP-L, PCA/GMP-H, and PCA/GAMP-H were constructed by transferring pYYDT and its derivates, pYhjH (26), pYedQ (26), and pGacA, into WT *G. sulfurreducens* PCA via electroporation. The construction of pGacA is provided in the supplemental material. All *G. sulfurreducens* strains were cultured anaerobically at 30°C in a minimal medium (referred to as NB) with 20mM acetate as the electron donors and 40mM fumaric acid as the electron acceptors (24). The NB medium contained KCl (0.38 g L^−1^), NH_4_Cl (0.2 g L^−1^), NaH_2_PO_4_·H_2_O (0.069 g L^−1^), CaCl_2_·2H_2_O (0.04 g L^−1^), and MgSO_4_· 7H_2_O (0.2 g L^−1^). The medium was adjusted to pH 6.8 with 2 g L^−1^ NaHCO_3_ and was stripped of dissolved oxygen (O_2_) by equilibrating with an O_2_-free mixture of N_2_ and CO_2_ gas (80:20, vol:vol). The media were then autoclaved. When required, the medium was supplemented with 200 µg mL^−1^ kanamycin (Km). The presence of pYYDT and its derivates in *G. sulfurreducens* PCA cells was monitored by extracting plasmids for gel electrophoresis analysis following our experiments.

### Preparation and characterization of ferric iron minerals

Ferrihydrite and goethite were prepared by the protocols established previously (see the supplemental methods) (31). The crystal structure of the synthesized minerals was confirmed using X-ray diffraction (XRD) with a Bruker AXS GmbH D8-Focus-Power Diffraction System (Bruker Corporation, Billerica, MA, USA), and the morphology was observed using a HITACHI SU8010 scanning electron microscope (SEM) (Hitachi, Chiyoda, Japan) (Fig. S1). The content of iron in the ferrihydrite, goethite, and lepidocrocite was determined to be 581.88, 715.45, and 579.83 g kg^−1^, respectively, by using an Optima 8000 inductively coupled plasma optical emission spectrometry (ICP-OES) (PerkinElmer, USA).

### Iron reduction kinetics

The mineral-coated glass slides were prepared by the protocols established previously (32). Briefly, three ferric iron minerals were prepared as a suspension containing 50 mM Fe(III). A 600 µL aliquot of suspension was deposited onto glass slides with an area of 1.8 cm^2^, ensuring that each slide contains an equal amount of ferric iron. The mineral-coated glass slides were dried at room temperature and then placed in the 12-well plates (Jet Biofil, Guangdong, China) containing 2 mL NB medium with 20 mM acetate as the electron donors. Diluted overnight cultures of *G. sulfurreducens* strains, at final turbidity of optical density at 600 nm (OD_600nm_) ~0.1, were inoculated in the 12-well plates. The cultures were anaerobically incubated at 30°C without shaking. The *G. sulfurreducens* cells formed biofilms on mineral-coated glass slides and reduced Fe(III) to ferrous iron [Fe(II)] (Fig. S2). To ensure experimental reproducibility, multiple reaction setups were prepared at the beginning of the experiment. At each time point, three independent reactions were allocated as biological replicates for the quantification of protein and iron concentration. After data collection, these reactions were discarded, and the remaining setups proceeded to subsequent time points. At predetermined time intervals, the concentrations of total Fe(II) and total iron were measured using the ferrozine method and were analyzed with a Spectramax 190 microplate reader (Thermo Fisher Scientific, Waltham, MA, USA) at 562 nm (33, 34). The total iron was measured using a reported method (35).

The increase in Fe(II) concentration exhibited a linear relationship with time during the initial stages of the iron reduction process, specifically from 0 to 5 days (R^2^ values ranging from 0.979 to 0.996), 0 to 12 days (R² values ranging from 0.947 to 0.987), and 0 to 8 days (R² values ranging from 0.879 to 0.985) for the reductions of ferrihydrite, lepidocrocite, and goethite, respectively. This linearity corresponds with characteristics typical of a zero-order reaction model and renders it particularly suitable for comparing Fe(III) reduction rates across different strains and ferric minerals. Therefore, the rate constants of Fe(III) reductions were calculated using the pseudo-zero-order model (36). At the end of the experiments, the morphology and composition of the secondary minerals were determined using XRD and SEM.

Statistical analyses were performed using GraphPad Prism (version 9.0.0). Each experiment was performed with at least three replicates. Data are presented as the mean ± standard deviation (SD). To evaluate statistical significance, a two-tailed Student’s *t*-test was employed. The *P* values are indicated as follows: NS (not significant) for *P* > 0.05, * for *P* < 0.05, ** for *P* < 0.01, and *** for *P* < 0.001.

### Biofilm images and biomass measurement

Biofilm formation on the nonconductive surfaces was investigated using a well-plate method (37). Similar to biofilm formation on the mineral-coated glass slide, the cultures of *G. sulfurreducens* strains were inoculated into 24-well plates containing 1 mL NB medium with 20 mM acetate and 40 mM fumaric acid as the electron donors and receptors, respectively. The cultures were incubated without shaking under anaerobic conditions at 30°C for different durations. Subsequently, a biofilm assay based on the crystal violet staining method was performed (26, 38).

The biofilms grown on the mineral-coated glass slides were stained with a fluorescent nucleic acid stain SYTO 9 (2.5 μM) and were imaged under confocal laser scanning microscopy (CLSM, Leica Microsystems CMS GmbH) in the State Key Laboratory of Biogeology and Environmental Geology, China University of Geosciences (Wuhan). The images were obtained at laser wavelengths of 488 and 561 nm and were processed using Leica LAS X software. The biomass attached to the slides was determined using a previously established method (37, 38). In brief, the slides were placed into 50 mL tubes containing 5 mL of 0.2 mM NaOH. The tubes were incubated at 96°C for 1 hour, with gentle agitation throughout the process to ensure biofilm cell lysis. Afterward, the slides were removed, and the resulting lysates were centrifuged at 20,000 × *g* for 30 minutes. A 5 µL aliquot of the supernatant was then used to measure the total protein concentration using the Qubit protein assay kit and a Qubit fluorometer (Thermo Fisher Scientific, Waltham, MA, USA), with bovine serum albumin (BSA) used as a standard.

### RNA sequencing and data analysis

Bacterial cells were harvested from the biofilms formed on ferrihydrite-coated glass slides at maturation stages. The HiPure Universal RNA Mini Kit (Magen, Guangzhou, China) was utilized for total RNA extraction. The extracted RNA was examined on 1% agarose gels for quality evaluation. Quantification of RNA was carried out using a Qubit 3.0 (Thermo Fisher Scientific, Waltham, MA, USA). The details for gene expression analysis are provided in the supplemental material.

### Quantitative PCR (qPCR)

The differential expression of *c*-Cyt genes obtained in RNA-seq analysis was validated using qPCR. The primers used in this study are listed in Table S2. The RNA samples were used to synthesize cDNA by using a cDNA synthesis kit (Fermentas Life Sciences, Glen Burnie, MD, USA). The resultant cDNA was used in qPCR reactions on an ABI StepOnePlus system (Life Technologies, Foster City, CA, USA).

### Construction of deletion mutants

The gene-deletion mutants Δ1996 (i.e., without GSU1996 gene), Δ2513 (i.e., without GSU2513 gene), Δ2808 (i.e., without GSU2808 gene), and Δ3615 (i.e., without GSU3615 gene) were constructed by following previously published procedures (39). The primers used in the construction of the deletion mutants are listed in Table S2. To generate the deletion mutants, the chromosomal regions located either upstream or downstream of the target were amplified. The spectinomycin (Spe) resistance gene was amplified using a primer set S-F/S-R. Subsequently, overlap extension PCR was employed to fuse the upstream region of the target gene, the s*pe* gene, and the downstream region of the target gene using external forward and reverse primers. The resulting fused fragments were then transferred into electrocompetent cells of *G. sulfurreducens* via electroporation. The deletion mutants were verified with PCR and DNA sequencing.

## RESULTS

### Biofilm formation of four *G. sulfurreducens* strains on nonconductive surfaces

**Fig 1:**
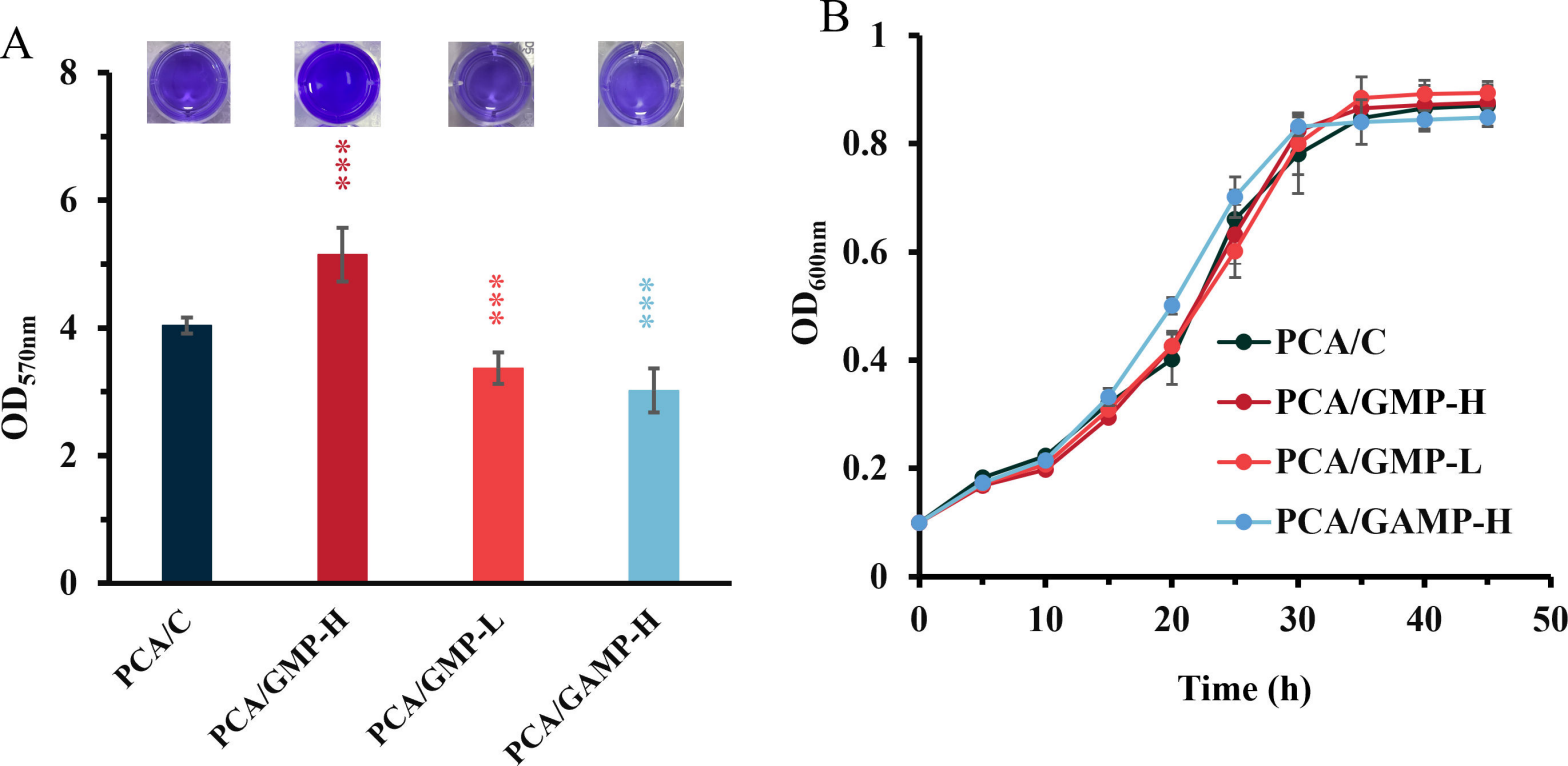
Biofilm formation of *G. sulfurreducens* cells on nonconductive surfaces. (**A**) Crystal-violet stained biomass of 72-hour-old biofilms formed by PCA/GMP-H, PCA/GMP-L, PCA/GAMP-H, and the control strain PCA/C on plastic surfaces (*n* = 6 independent samples). (**B**) Cell growth of four *G. sulfurreducens* strains in the medium using fumarate as electron acceptors (*n* = 3 independent samples). Data are shown as the mean ± SD. A two-sided Student’s *t*-test was used to analyze the statistical significance (***P < 0.001).

In our previous investigation, we constructed the PCA/GMP-L (low c-di-GMP), PCA/GMP-H (high c-di-GMP), and PCA/C (intermediate c-di-GMP) by transferring pYhjH containing a c-di-GMP hydrolase gene *yhjH*, pYedQ containing a c-di-GMP synthase gene *yedQ*, and an empty vector, respectively (Table S1). Here, we engineered the strain PCA/GAMP-H by overexpressing an endogenous cGAMP synthase GacA in *G. sulfurreducens*. The cGAMP level in PCA/GAMP-H was higher than that in the control strain PCA/C (62.61 ± 4.39 vs 45.76 ± 1.58 pg mg^−1^ total protein; Fig. S3). The *G. sulfurreducens* strains exhibited varying abilities to form biofilms on the cell culture plates over a 72 hour incubation in the medium using fumarate as electron acceptors (Fig. 1A). The absorbance of crystal violet (OD_570nm_) extracted from stained biofilms grown on nonconductive surfaces by the control strain PCA/C was lower than that of PCA/GMP-H, while being higher than that of both PCA/GMP-L and PCA/GAMP-H. The four strains did not show a significant difference in the growth on fumarate (Fig. 1B), suggesting that the significant difference in biofilm biomass was not caused by cell growth.

### Biofilm formation of four *G. sulfurreducens* strains on Fe(III)-containing mineral surfaces

**Fig 2:**
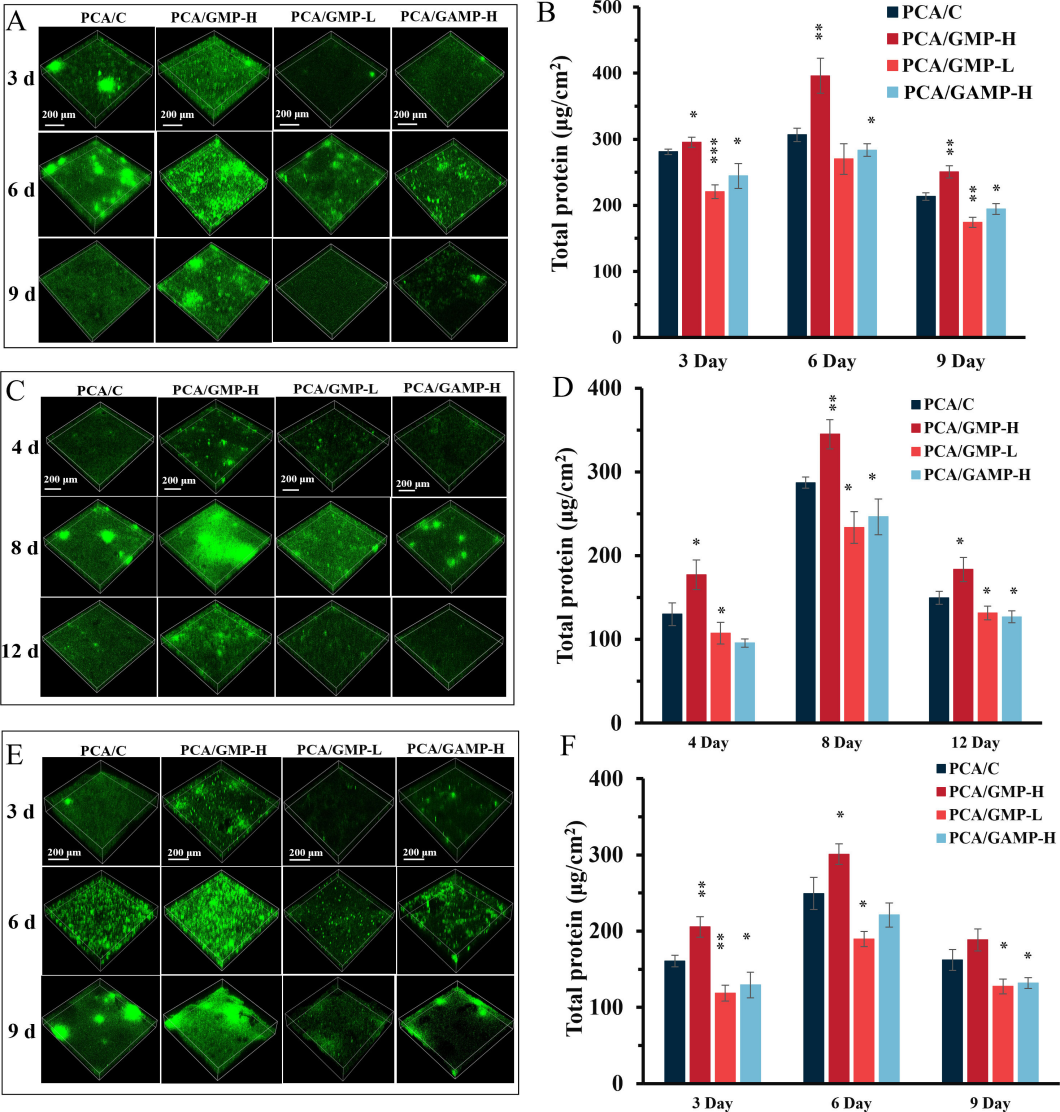
Biofilm formation of *G. sulfurreducens* cells on Fe(III)-containing minerals. CLSM images of biofilms on glass slides coated by (**A**) ferrihydrite, (**C**) lepidocrocite, and (**E**) goethite. The attached biomass of *G. sulfurreducens* biofilms on (**B**) ferrihydrite, (**D**) lepidocrocite, and (**F**) goethite-coated glass slides. CLSM images show the top and side views of biofilms. Scale bar, 200 µm. Data in panels **B, D, and F** are shown as the mean ± SD (*n* = 3 independent samples). A two-sided Student’s *t*-test was used to analyze the statistical significance (*P < 0.05, **P < 0.01, and ***P < 0.001).

The four *G. sulfurreducens* strains were inoculated into a system where Fe(III)-containing minerals were deposited on glass slides to serve as electron acceptors. The capabilities of *G. sulfurreducens* strains to form biofilms on the surfaces of Fe(III) oxide-coated glass slides were assessed. Observation of stained biofilms using CLSM showed a rise and subsequent decline in biofilm thickness on ferrihydrite-coated surfaces from 3 to 9 days after inoculation for all *G. sulfurreducens* strains (Fig. 2A), which represents a typical biofilm life cycle including attachment, maturation, and dispersal (25). However, there was a significant variation in the ability of biofilm formation among the *G. sulfurreducens* strains used. The PCA/GMP-H strain, characterized by a higher concentration of c-di-GMP, exhibited the formation of thicker biofilms. In contrast, both the PCA/GMP-L strain, which has a lower level of c-di-GMP, and the PCA/GAMP-H strain with elevated levels of cGAMP produced thinner biofilms when compared to the control strain PCA/C (Fig. 2A). For example, the average thickness of 6-day-old PCA/C biofilms was 52.08 ± 9.81 µm, which is thinner than 6-day-old PCA/GMP-H biofilms (74.51 ± 1.94 µm) but thicker than 6-day-old biofilms formed by PCA/GMP-L (34.41 ± 3.16 µm) and PCA/GAMP-H (36.37 ± 4.37 µm) (Fig. S4A). The biomass that adhered to the slides was consistent with the observed thickness (Fig. 2B). However, the biomass of PCA/C planktonic cells in the supernatants is significantly greater than that of PCA/GMP-H yet lower than those of PCA/GMP-L and PCA/GAMP-H (Fig. S5). This observation suggests that a decrease in c-di-GMP levels or an increase in cGAMP levels may reduce biofilm formation or promote the dispersal of *G. sulfurreducens* biofilms.

Similar trends were observed in the systems with lepidocrocite and goethite, where the thickness of *G. sulfurreducens* biofilms initially increased, followed by a subsequent decrease (Fig. 2C and E). The average thickness of mature PCA/C biofilms was 52.89 ± 2.63 µm on lepidocrocite and 53.24 ± 3.08 µm on goethite surfaces, which are lower than the thicknesses of 69.58 ± 11.55 µm and 73.24 ± 4.39 µm observed for PCA/GMP-H biofilms on lepidocrocite and goethite, respectively. However, the average thickness of PCA/C biofilms is greater than those formed by PCA/GMP-L (35.51 ± 6.61 µm on lepidocrocite and 36.38 ± 1.96 µm on goethite) and PCA/GAMP-H (38.33 ± 3.03 µm on lepidocrocite and 40.48 ± 3.67 µm on goethite) (Fig. S4B and C). Biofilm biomass increased in the order of PCA/GMP-L (~PCA/GAMP-H) < PCA/C < PCA/GMP-H (Fig. 2D and F). Although biofilm biomass increased in the order of PCA/GMP-L (~PCA/GAMP-H) < PCA/C < PCA/GMP-H in all systems with different Fe(III)-containing minerals, the biofilm biomass for all strains in the ferrihydrite system was higher than that in the lepidocrocite or goethite system. This could be attributed to the higher bioavailability of ferrihydrite.

### Reduction of ferric minerals by *G. sulfurreducens* biofilms

**Fig 3:**
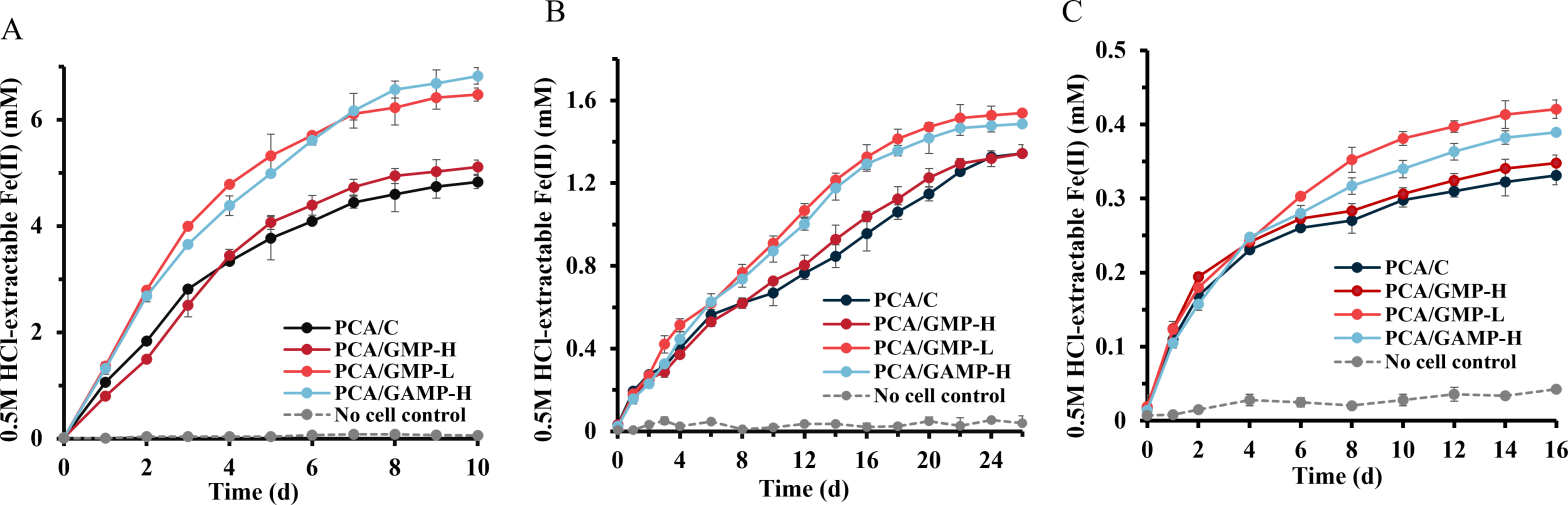
The production of Fe(II) resulting from the bioreduction of (**A**) ferrihydrite, (**B**) lepidocrocite, and (**C**) goethite by PCA/GMP-L, PCA/GMP-H, PCA/GAMP-H, and PCA/C. No significant Fe(III) reduction (<1% of the total iron) was observed in the abiotic controls prepared under the same conditions but without inoculation. The error bars indicate the standard deviation of triplicate samples.

As shown in Fig. 3, all *G. sulfurreducens* strains were able to reduce the ferric minerals. Among the different ferric minerals tested, we observed significantly more bioreduction of ferrihydrite (32.18%–45.48% of total Fe reduced) compared to those using lepidocrocite and goethite as electron acceptors (8.95%–10.26% for lepidocrocite and 2.21%–2.80% for goethite) (Table 1). The Fe(II) concentrations determined in the abiotic controls were only 0.28%–0.53% of the total iron, indicating no iron reduction in the absence of cultures (Fig. 3).

**TABLE 1:** Summary of iron reduction rates, extents, and distribution of secondary minerals under the investigated conditions

Culturing conditions	Rate (*k*) (mmol·L^−1^·day^−1^)*^a^*^,*b*^	Fraction of Fe(III) substrate reduced (%)*^a^*^,*c*^	Identification of secondary minerals	
			XRD^*d*^	SEM
Ferrihydrite				
PCA/C	0.754 ± 0.081	32.2 ± 0.83	GR, Sid	GR, Sid
PCA/GMP-H	0.813 ± 0.026 (NS)^*e*^	34.1 ± 0.90	GR, Sid	GR, Sid
PCA/GMP-L	1.064 ± 0.046*	43.1 ± 1.50	GR, Sid	GR, Sid
PCA/GAMP-H	0.998 ± 0.010*	45.5 ± 1.02	GR, Sid	GR, Sid
Lepidocrocite				
PCA/C	0.064 ± 0.004	9.0 ± 0.26	/^*f*^	Sid, Viv, GR
PCA/GMP-H	0.067 ± 0.002 (NS)	9.0 ± 0.28	/	Sid, Viv, GR
PCA/GMP-L	0.089 ± 0.003**	10.3 ± 0.45	/	Sid, Viv, GR
PCA/GAMP-H	0.083 ± 0.002**	9.9 ± 0.27	/	Sid, Viv, GR
Goethite				
PCA/C	0.034 ± 0.002	2.2 ± 0.08	/	Sid
PCA/GMP-H	0.035 ± 0.001 (NS)	2.3 ± 0.07	/	Sid
PCA/GMP-L	0.044 ± 0.004*	2.8 ± 0.13	/	Sid
PCA/GAMP-H	0.040 ± 0.001*	2.6 ± 0.02	/	Sid

^*a*^ The uncertainties indicate the standard deviation of triplicate samples.

^*b*^ The rate constants of ferrihydrite, lepidocrocite, and goethite reductions were calculated using the pseudo-zero-order model for the data sets for 5, 12, and 8 days, respectively.

^*c*^ Calculated as the ratio of 0.5 M HCl-extractable Fe(II) to the starting Fe(III) concentration in the reactor.

^*d*^ Abbreviations: GR, green rust; Sid, siderite; Viv, vivianite.

^*e*^ Statistical significance was determined using two-sided Student’s *t*-test for comparing the groups of cyclic dinucleotide-perturbed strains with the control strain. *P* values are reported using the following symbolic representation: NS (no significance) *P* > 0.05, *P < 0.05, **P < 0.01.

^*f*^ “/” signifies not detected.

The rates of Fe(III) reduction varied significantly among the four *G. sulfurreducens* strains. At 5 days of ferrihydrite reduction, the Fe(III) reduction rates of PCA/GMP-L and PCA/GAMP-H, both forming a relatively thinner biofilm, were comparable (1.064 ± 0.046 mmol·L^−1^·day^−1^ for PCA/GMP-L and 0.998 ± 0.010 mmol·L^−1^·day^−1^ for PCA/GAMP-H), which was significantly faster than that of the control strain PCA/C (0.754 ± 0.081 mmol·L^−1^·day^−1^; Table 1). However, PCA/GMP-H, which formed a thicker biofilm, exhibited a similar trend in Fe(II) production as PCA/C (Fig. 3A and Table 1). Furthermore, we observed that the concentrations of Fe(II) and total iron in the supernatants did not differ significantly (Fig. S6), indicating that the ferric iron on the slides did not detach into the supernatants. Notably, the concentration of Fe(II) in the supernatants showed no significant variation among the four *G. sulfurreducens* strains; however, there was a significant difference in total Fe(II) concentration (Fig. 3A), which includes both Fe(II) associated with minerals and that present in the supernatants. These findings further suggest that iron reduction occurs at the interface between minerals and biofilms.

We observed similar Fe(III) reduction rates for lepidocrocite or goethite by the four *G. sulfurreducens* strains during the first 6 days of reduction (Fig. 3B and C). After the first 6 days, PCA/GMP-L and PCA/GAMP-H displayed a faster and more extensive iron reduction compared to PCA/C and PCA/GMP-H (Fig. 3 and Table 1). The Fe(III) reduction of PCA/GAMP-H was similar to that of PCA/GMP-L in ferrihydrite and lepidocrocite systems but slightly slower than that of PCA/GMP-L in the goethite system (Fig. 3). This difference could be attributed to the distinct physiochemical characteristics of different ferric minerals, such as their redox potentials. PCA/GMP-L biofilms exhibit the capability to effectively facilitate electron transfer in the presence of Fe(III)-containing minerals characterized by low redox potentials.

Spectroscopic and microscopic methods were used to analyze the formation of secondary minerals. Green rust and siderite were detected as secondary minerals by XRD and SEM after 13 days of ferrihydrite reduction by all four *G. sulfurreducens* strains used (Table 1; Fig. S7). Although SEM analyses showed possible formation of siderite, vivianite, and/or green rust after reduction of goethite or lepidocrocite (Fig. S8 and S9), XRD analyses could not confirm these potential secondary minerals due to low abundance or poor crystallinity. The results indicate that no difference in the formation of secondary minerals was observed for the same ferric minerals among the four *G. sulfurreducens* strains.

### Differential gene expression in biofilm cells of engineered *G. sulfurreducens* strains

**Fig 4:**
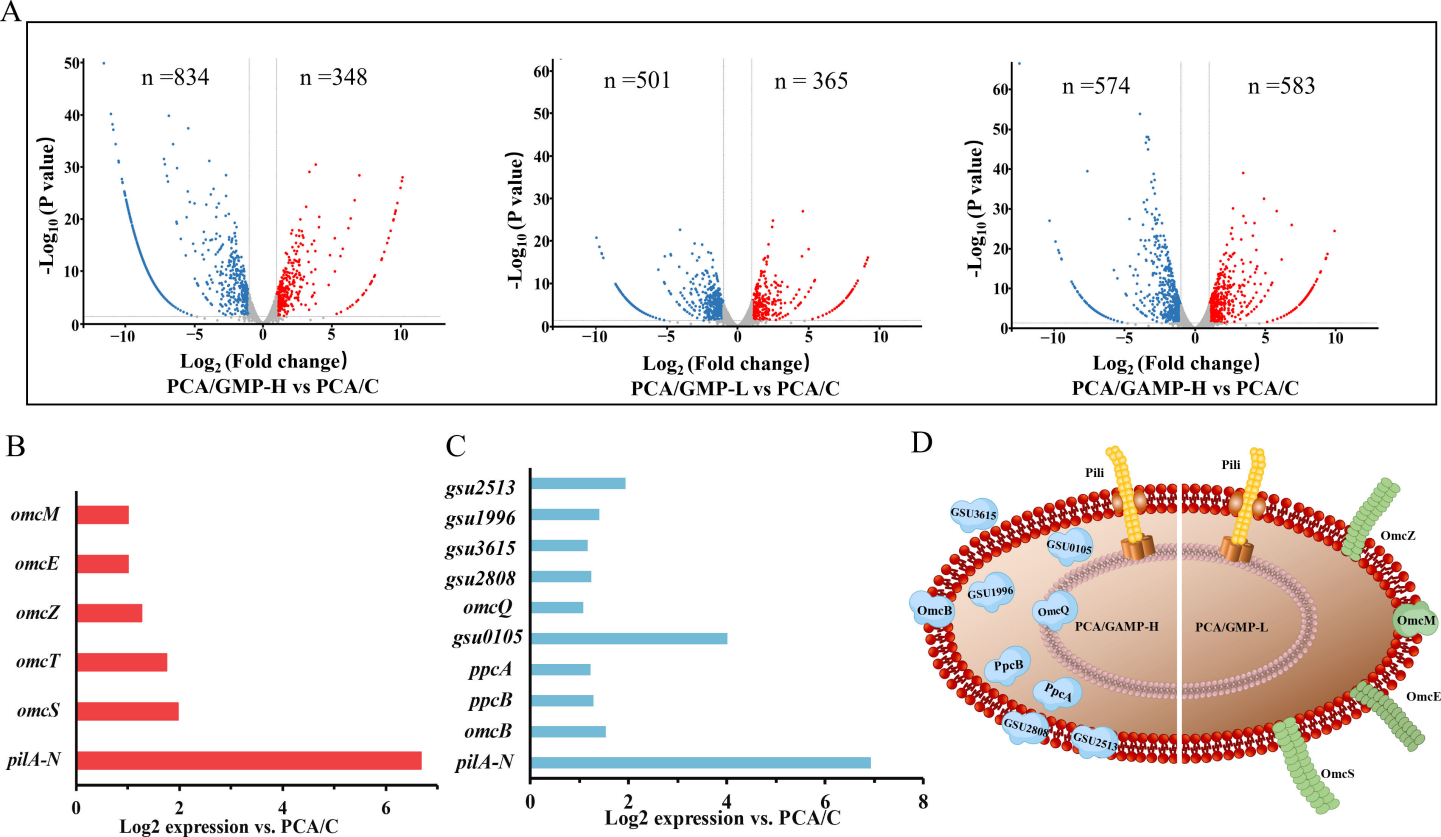
The gene expression in *G. sulfurreducens* biofilm cells. (**A**) Volcano plots for differential gene expression analysis between PCA/GMP-H, PCA/GMP-L, or PCA/GAMP-H and the control strain PCA/C. Comparison of the expression of electron transfer genes in the control strain PCA/C vs (**B**) PCA/GMP-L and (**C**) PCA/GAMP-H. (**D**) The predicted subcellular locations of EET-involved proteins upregulated in the cells of PCA/GAMP-H and PCA/GMP-L biofilms.

To further explore the underlying mechanism of biofilm formation and Fe(III) reduction, we conducted RNA-seq-based transcriptomics analysis. Total RNA was isolated from the cells of 6-day-old biofilms of *G. sulfurreducens* strains formed on the glass slides coated with ferrihydrite. The RNA seq generated a total of 3,366 reads. Compared to the control strain PCA/C, expression analysis revealed that 1,182 genes (348 upregulated and 834 downregulated), 866 genes (365 upregulated and 501 downregulated), and 1,165 genes (585 upregulated and 574 downregulated) were differentially expressed in PCA/GMP-H, PCA/GMP-L, and PCA/GAMP-H, respectively (Fig. 4A), using a significance threshold of *P* value < 0.05 with a fold change of >2. These accounted for approximately 26% to 35% of the genes in the *G. sulfurreducens* genome, indicating perturbations in intracellular c-di-GMP and cGAMP levels triggered significant global changes in gene expression.

The overall rate of electron transfer from biofilms to ferric minerals primarily could depend on two key factors: (i) the capacity for biofilm formation, which determines the number of metabolically active cells contributing Fe(III) reduction; and (ii) the conductivity of the biofilm, which influences the efficiency of electron transfer. Transcriptomics analysis showed differential expression of genes involved in biofilm formation and electron transfer in engineered *G. sulfurreducens* strains.

#### Expression of genes involved in biofilm formation

Compared to the control strain PCA/C, a low expression of biofilm-associated genes was found in the strains PCA/GMP-L and PCA/GAMP-H, forming a thinner biofilm (Fig. S10). These findings are consistent with the observed reduction of biofilm formation when c-di-GMP levels were decreased or a cGAMP level was increased in *G. sulfurreducens* (Fig. 1 and 2). In contrast, these genes were upregulated in PCA/GMP-H that formed a thicker biofilm. This probably contributed to enhanced biofilm formation.

#### Expression of the genes for EET

mRNA levels of the genes for extracellular pilin protein PilA-N increased in both strains PCA/GMP-L and PCA/GAMP-H that formed thin biofilms on mineral surfaces, as compared to that in PCA/C (Fig. 4B and C). mRNA levels of the genes for the outer membrane *c*-Cyt OmcM and extracellular *c*-Cyts OmcS, OmcT, OmcZ, and OmcE were upregulated in PCA/GMP-L cells but not in PCA/GAMP-H cells (Fig. 4B). On the other hand, the other nine *c*-Cyts-encoding genes were upregulated only in PCA/GAMP-H cells (Fig. 4C). The subcellular localizations were predicted as follows: OmcQ was predicted to localize to the inner membrane; GSU0105, GSU1996, PpcA, and PpcB were predicted to localize to the periplasmic space; OmcB, GSU2513, and GSU2808 were predicted to localize to the outer membrane; and GSU3615 was predicted to be extracellular (Table S3; Fig. 4D). Most of the *c*-Cyts upregulated in PCA/GMP-L and PCA/GAMP-H have been previously reported to be involved in Fe(III) reduction by *G. sulfurreducens*. The higher expression level of genes encoding EET proteins in PCA/GMP-L and PCA/GAMP-H cells was further confirmed using qPCR (Fig. S11), which was consistent with the RNA-seq results.

### Involvement of GSU1196 and GSU2513 in the reduction of Fe(III)-containing minerals by *G. sulfurreducens* biofilms

**Fig 5:**
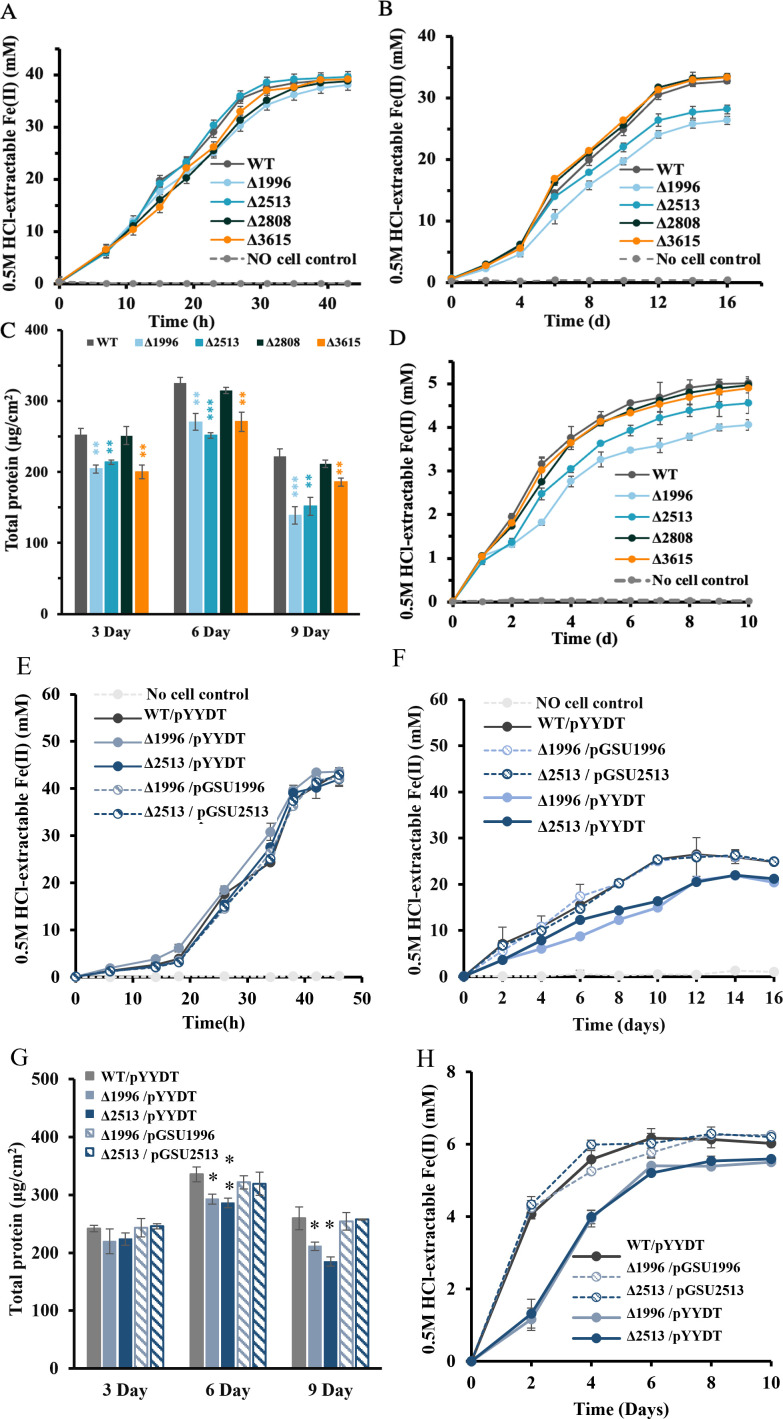
Characterizations of the genes involved in the EET of *G. sulfurreducens*. The reduction of (**A**) Fe(III) citrate and (**B**) ferrihydrite by planktonic cells of gene-deletion mutants. (**C**) The biomass of 3-, 6-, and 9-day-old biofilms formed by gene-deletion mutants on ferrihydrite-coated glass slides. (**D**) The production of Fe(II) resulting from ferrihydrite bioreduction by the biofilms of gene-deletion mutants. The reduction of (**E**) Fe(III) citrate and (**F**) ferrihydrite by planktonic cells of Δ1996, Δ2513, and their complementation strains. (**G**) The biomass of 3-, 6-, and 9-day-old biofilms formed by Δ1996, Δ2513, and their complementation strains on ferrihydrite-coated glass slides. (**H**) The production of Fe(II) resulting from ferrihydrite bioreduction by the biofilms of Δ1996, Δ2513, and their complementation strains. The error bars indicate the standard deviation of triplicate samples. A two-sided Student’s *t*-test was used to analyze the statistical significance (**P < 0.01 and ***P < 0.001).

The *c*-Cyts GSU1996, GSU2513, GSU2808, and GSU3615 were upregulated in PCA/GAMP-H biofilm cells. To assess their roles in the EET between *G. sulfurreducens* biofilm and ferric minerals*, gsu1996*, *gsu2513*, *gsu2808*, and *gsu3615* genes were individually deleted. Deletions of these genes had no impact on the bacterial growth on soluble electron acceptors such as fumarate (Fig. S12) and Fe(III) citrate (Fig. 5A). However, the deletion of *gsu1996* or *gsu2513* resulted in a reduced ability of planktonic *G. sulfurreducens* cells to reduce ferrihydrite (Fig. 5B), indicating their involvement in the EET of *G. sulfurreducens*.

We further tested the ability of these gene-deletion mutants to form biofilms on ferrihydrite-coated glass slides. The biofilm biomass of Δ1996, Δ2513, and Δ3615 was significantly lower than that of WT *G. sulfurreducens* (Fig. 5C). The Fe(III) reduction was monitored during the formation of *G. sulfurreducens* biofilms. The Fe(III) reduction rates of Δ1996 and Δ2513 were 0.652 ± 0.034 mmol·L^−1^·day^−1^ and 0.726 ± 0.006 mmol·L^−1^·day^−1^, respectively, which were significantly slower than that of WT (0.842 ± 0.031 mmol·L^−1^·day^−1^; Fig. 5D; Table S4). However, Δ2808 and Δ3615 exhibited a similar Fe(II) production trend with WT (Fig. 5D; Table S4). As shown in Fig. 5E, no significant difference was observed in the reduction of Fe(III) citrate among WT *G. sulfurreducens* containing an empty vector, Δ1996 containing an empty vector, Δ2513 containing an empty vector, and their respective complementation strains. However, the introduction of *gsu1996* and *gsu2513* successfully restored the abilities of Δ1996 and Δ2513 to reduce ferrihydrite in planktonic states, form biofilms on mineral surfaces, and facilitate biofilm-mediated ferrihydrite reduction (Fig. 5F through H). These findings further confirm the critical roles of GSU1996 and GSU2513 in the reduction of ferric minerals.

## DISCUSSION

For *Geobacter* spp., the utilization of insoluble electron acceptors (e.g., electrodes and minerals) requires direct contact to facilitate electron transfer by forming biofilms (16). EET in *Geobacter* biofilms has been extensively studied in bioelectrochemical systems using electrodes as electron acceptors (40). However, the findings from electrochemistry studies are challenging to describe the process of Fe(III)-containing mineral reduction by *Geobacter* biofilms in natural systems. To the best of our knowledge, the present study is the first to systematically investigate *G. sulfurreducens* biofilm formation on Fe(III)-containing mineral surfaces and subsequently to mechanistically examine the role of biofilm formation in Fe(III) oxide reduction activity.

Previous research showed that *G. sulfurreducens* forms electroactive biofilms tens of micrometers away from the electrode surface, with the thickness of the biofilm within a specific range directly correlating to the generated current (41, 42). The enhancement of biofilm formation on anodes through genetic engineering of pili (19) and flagella (21), or increased polysaccharide biosynthesis (20), facilitates the EET in *G. sulfurreducens*. In our previous study, we also observed that a thicker anode biofilm formed by PCA/GMP-H produced a higher current compared to that formed by the control strain PCA/C (26). As observed in our previous study (26), PCA/GMP-H formed a thicker biofilm on both nonconductive surfaces and the surfaces of Fe(III)-containing minerals than PCA/C in this investigation (Fig. 1 and 2). However, PCA/GMP-H biofilms exhibited a similar rate of ferrihydrite, lepidocrocite, and goethite reduction as PCA/C biofilms (Fig. 3; Table 1). The observed discrepancy between biofilm thickness and iron reduction could potentially be attributed to diffusion limitations of nutrients and buffers within thicker biofilms. However, it is important to note that mass transfer limitations for nutrients and buffers do not occur in *Geobacter* biofilms with a thickness of up to 80 µm (43). Because the thickness of PCA/GMP-H biofilms formed on all minerals in our study is less than 80 µm (Fig. S4), it is unlikely that mass transfer limitations are a critical factor in our system. The different observations between the previously reported bioelectrochemical systems and the Fe(III) oxide systems in this study may be due to the much lower potential redox of Fe(III) oxides, which presents a thermodynamic challenge for electron transfer in relatively thick *G. sulfurreducens* biofilms compared to those on anodes (44).

Although the strains PCA/GMP-L and PCA/GAMP-H formed thinner biofilms on Fe(III) oxides (Fig. 2), they exhibited a faster rate of Fe(III) reduction (Fig. 3 and Table 1). A similar observation was reported in our previous study (26), indicating that the thin but conductive anode biofilm formed by PCA/GMP-L produced a high electrical output. In this investigation, transcriptomic analyses revealed upregulated expressions of matrix-associated electron carriers such as the PilA pilin subunit and *c*-Cyts OmcS, OmcZ, OmcE, and OmcT in PCA/GMP-L (Fig. 4B). The results are consistent with a previous study (26). The deletion of these genes diminished the ability of *G. sulfurreducens* to reduce Fe(III) oxides (24), suggesting their important roles in the EET between *G. sulfurreducens* and metal oxides. Additionally, the expression of the outer membrane *c*-Cyt OmcM was also upregulated in PCA/GMP-L cells. A more abundant gene transcript of OmcM was observed during Fe(III)-reducing conditions in the previous study (42). In PCA/GAMP-H cells, we observed upregulated expressions of *c*-Cyts, including OmcQ, GSU0105, PpcB, PpcA, and OmcB (Fig. 4C). Their direct involvement in the EET of *Geboacter* were identified by omics-based and genetic studies (33, 45, 46). The highly expressed proteins involved in EET may confer high conductivity to *G. sulfurreducens* biofilms, resulting in high activity of Fe(III) oxide reduction.

Our results also showed the increased expressions of *c*-Cyts GSU1996, GSU2513, GSU2808, and GSU3615 in the thin biofilms formed by PCA/GAMP-H, as well as provided the evidence of involvements of GSU1996 and GSU2513 in the EET of *G. sulfurreducens*. The deletion of genes for GSU1996 and GSU2513 impaired ferrihydrite reduction by both planktonic cells and biofilms of *G. sulfurreducens* (Fig. 5B and D). The predicted subcellular locations were periplasmic for GSU1996 and the outer membrane for GSU2513. Thermodynamic and kinetic analyses of GSU1996, a dodecaheme cytochrome comprising four triheme domains (47), demonstrated that a heme located at the C-terminal effectively accepts electrons from redox partners (48). The investigation into the interactions between GSU1996 and the periplasmic cytochrome PpcA further supported its role in electron transfer (49). Additionally, an upregulation of GSU2513 in *G. sulfurreducens* was found during Pd(II) reduction (50). These findings highlight the involvement of both GSU1996 and GSU2513 in EET processes. Our study provides direct evidence for their roles in EET, particularly in iron reduction. The extracellular *c*-Cyt GSU3615 reduced biofilm formation (Fig. 5C), while reduced biofilms did not impair ferrihydrite reduction (Fig. 5D). This suggests that the extracellular protein, responsible for biofilm formation but not for *G. sulfurreducens* EET, is not essential for Fe(III) reduction by *G. sulfurreducens* biofilms. All the aforementioned results consistently indicate that the proteins involved in *G. sulfurreducens* EET are decisive variables for the high activity of Fe(III) oxide reduction by *G. sulfurreducens* biofilms.

Collectively, this study used Fe(III) mineral-coated slides to mimic microbe–iron interactions in natural environments, specially examining *G. sulfurreducens* biofilm formation on minerals and biofilm-mediated Fe(III) reduction. The study revealed that thin biofilms exhibiting elevated levels of EET proteins displayed a significantly enhanced Fe(III) reduction activity. In contrast, biofilms lacking electron transfer proteins showed reduced activity. This study provides experimental evidence to support the hypothesis that *Geobacter* spp. must organize electron transfer proteins to extend outward in order to access Fe(III) located several microns beyond their outer membrane (44). Given that *Geobacter* spp. employ different proteins for electron transfer depending on the redox potential of various Fe(III) oxides (51), further study on the identification of crucial electron transfer proteins for different Fe(III) oxide reductions by *Geobacter* biofilms will provide clues to understand what controls the competitiveness of *Geobacter* in the environment. The findings in this research enhance our understanding of biofilm-mediated Fe(III) oxide reduction, thereby improving knowledge of biogeochemical processes that impact iron transformations and the fate of other elements such as carbon, nitrogen, sulfur, and arsenic (52).

## Data Availability

The raw data have been deposited in the National Center for Biotechnology Information (NCBI) under BioProject number PRJNA1143950.

## SUPPLEMENTAL MATERIAL

Supplemental material aem.00369-25-s0001.pdf Supplemental methods, Fig. S1 to S12, and Tables S1 to S4.

ASM does not own the copyrights to Supplemental Material that may be linked to, or accessed through, an article. The authors have granted ASM a non-exclusive, world-wide license to publish the Supplemental Material files. Please contact the corresponding author directly for reuse.
