# Supplementary material for: Extracellular electron transfer proteins contribute to reduction of ferric minerals by Geobacter biofilms
Source: Appl Environ Microbiol. 2025 Apr 9;91(5):e00369-25. doi: 10.1128/aem.00369-25 (PMC12094021; doi:10.1128/aem.00369-25)
Supplement: Supplemental material — Supplemental methods, Fig. S1 to S12, and Tables S1 to S4. [file aem.00369-25-s0001.pdf]

Supporting Information for

Extracellular Electron Transfer Proteins Contribute to the Reduction of Ferric Minerals by *Geobacter* Biofilms

Jiacheng Xu[a#], Wei Zhou[a#], Xi Han[a], Jian Liu[a], Yiran Dong[a, b], Yongguang Jiang[a], Yuhong Zhong[a], Liang Shi[a ,b, c, d*] and Yidan Hu[a*]

[a] School of Environmental Studies, China University of Geosciences, Wuhan 430074, Hubei, China

[b] State Key Laboratory of Geomicrobiology and Environmental Changes, China University of Geosciences, Wuhan 430074, Hubei, China

[c] Hubei Key Laboratory of Yangtze Catchment Environmental Aquatic Science, China University of Geosciences, Wuhan 430074, Hubei, China

[d] State Environmental Protection Key Laboratory of Source Apportionment and Control of Aquatic Pollution, Ministry of Ecology and Environment, China University of Geosciences, Wuhan 430074, Hubei, China

# These authors contributed equally to this work.

* Corresponding author

Email: Liang.shi@cug.edu.cn (L. Shi); huyidan@cug.edu.cn (Y. Hu)

No. of pages: 22

No. of Figures: 12 (S1, S2, S3, S4, S5, S6, S7, S8, S9, S10, S11, S12)

No. of Tables: 4 (S1, S2, S3, S4)

Supplementary materials and methods

The construction of PCA/GAMP-H. The cGAMP synthase gene *gacA* from *G. sulfurreducens* with a ribosome binding site (RBS) was amplified using a pair of primers, GacA-F and GacA-R. Then, the fragment of RBS-*gacA* was digested using restriction enzymes, XabI and PstI, and ligated with the vector pYYDT digested by SpeI and SbfI. The ligation products were transferred into *E. coli* DH5α through the heat shock method. The constructs were selected by colony polymerase chain reaction (PCR) using YYD-F and YYD-R2 and further verified to confirm the sequences by DNA sequencing. The sequence details for the primers are included in Supplementary Table 1. The plasmid of pGacA was electroporated into electrocompetent cells of *G. sulfurreducens*, generating the strains of PCA/pGacA. Plasmid isolation kit and PCR product purification kit were purchased from Thermo Fisher Scientific (Waltham, MA, USA). DNA sequencing was performed in The Beijing Genomics Institute (Wuhan, China). The intracellular concentration of cGAMP was measured using 2'3'-cGAMP ELISA Kit (Cayman, Michigan, USA).

Preparation and characterization of ferric iron mineral. Ferrihydrite [Fe_2O_3•$0.5H_2O$] and goethite [α-FeO(OH)] were prepared as followed. Ferrihydrite was prepared by titrating 0.5 M $FeCl_3$ to pH 7.5 by the dropwise addition of 1.0 M KOH with continuous mixing. Goethite was synthesized by aging ferrihydrite under alkaline conditions at 70°C for 60 h. Lepidocrocite was provided by Professor Edward J.O'loughlin (Argonne National Laboratory). Briefly, 30 g of $FeCl_2$•$4H_2O$ was dissolved in 900 mL of water and the resulting solution was filtered through a 0.2 μm nylon filter to remove any Fe(III) solids present. The pH of the solution was adjusted to 6.0 with 0.5 M NaOH and the resulting blue/green suspension was sparged with air. The pH of the suspension was maintained at pH 5.5-6.0 by the dropwise addition of 0.5 M NaOH until base consumption ceased (~1 h). The synthesis was conducted in a large > 4 L capacity desiccator. Subsequent to synthesis, all phases were repeatedly washed by

centrifugation and re-suspension in Milli-Q® $H2O$ (18.2 MΩ•cm), then dried at 60°C and ground to pass a 200-mesh sieve (ferrihydrite was washed, but not dried).

Measurement of iron concentration. When measuring total iron concentrations, an additional 200 μL of 1 M HCl was added into the pre-acidified samples which were then heated at 50°C to dissolve all the ferric iron oxides. Then, Fe(III) was reduced to Fe(II) using hydroxylamine hydrochloride before the total Fe(II) was measured using the ferrozine method. Fe(III) concentrations were calculated by subtracting the concentrations of ferrous iron from total iron. At the end of the experiments, the solid-associated Fe(II) [Fe(II)(s)] was calculated as the difference between acid extractable Fe(II) and Fe(II) (aq).

RNA-seq data analysis. Gene expression analysis was conducted using a cBot cluster generation system followed by sequencing on an Illumina Novaseq6000 platform with 150-bp paired-end reads generated using Magigene (China). Trimmomatic was employed to process raw data in FASTQ format into clean data. Clean reads were mapped to the NCBI Rfam database and rRNA sequences removed using Bowtie2. HTSeq-count provided read count and functional information for each gene based on mapping results while fragments per kilobase of transcript per million mapped reads were calculated for comparison of gene expression levels across different genes and experiments. The differential expression of genes between two groups was examined using edgeR with resulting p values adjusted via Benjamini and Hochberg's approach to determine false discovery rate. Transcripts exhibiting a fold-change greater than two and an adjusted p value less than 0.05 were identified as differentially expressed.

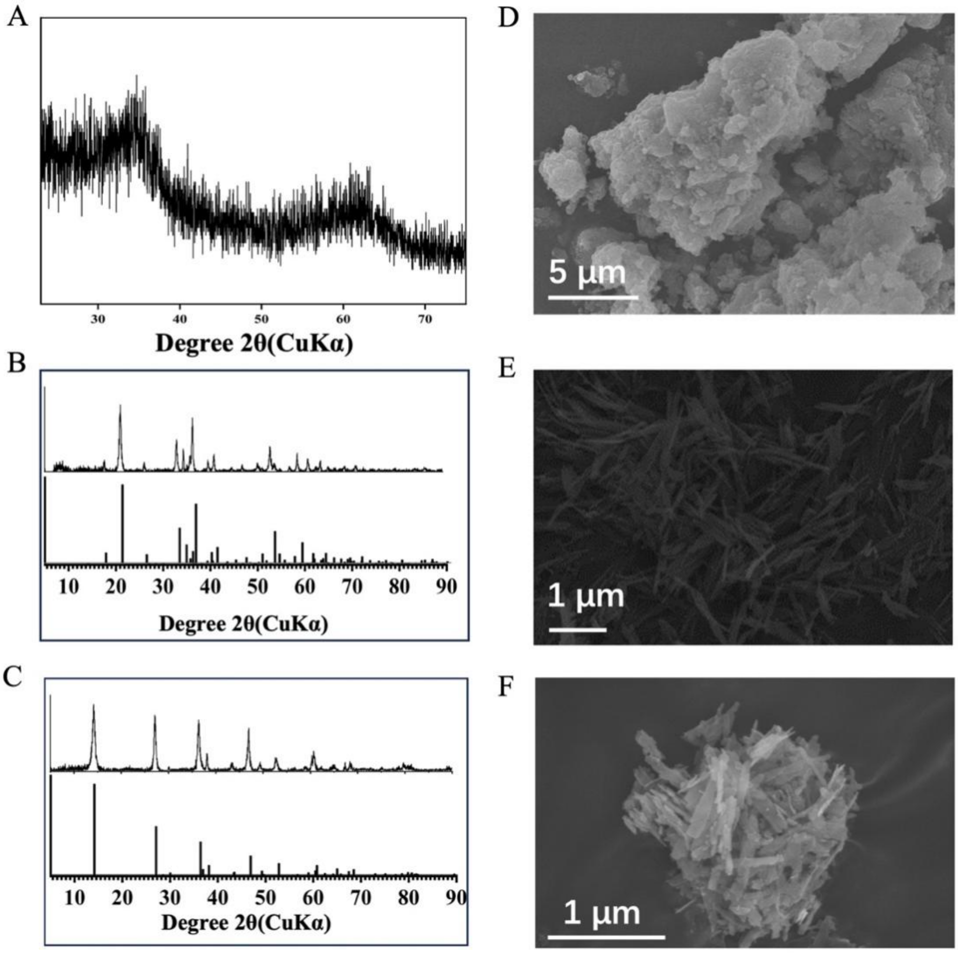

Fig. S1 XRD analysis of (A) ferrihydrite, (B) goethite and (C) lepidocrocite and SEM images of (D) ferrihydrite, (E) goethite and (F) lepidocrocite.

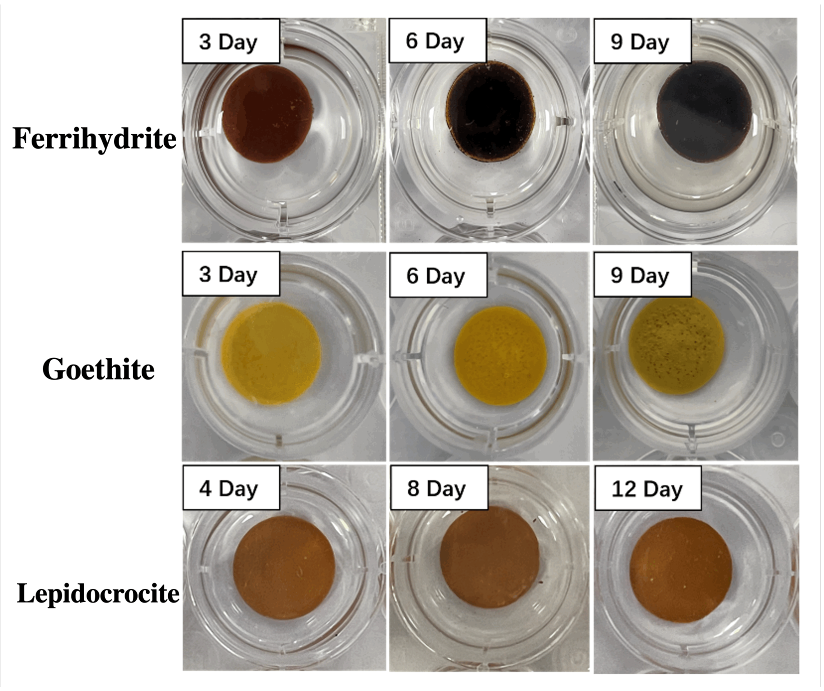

Fig. S2 Microbial iron reduction of ferrihydrite, goethite and lepidocrocite by wild type (WT) *G. sulfurreducens* PCA. The mineral-coated glass slides were placed in the 12-well plates containing 20 mM acetate as the electron donors. Diluted cultures of *G. sulfurreducens* strains were inoculated in the 12-well plates. The cultures were anaerobically incubated at 30 °C without shaking.

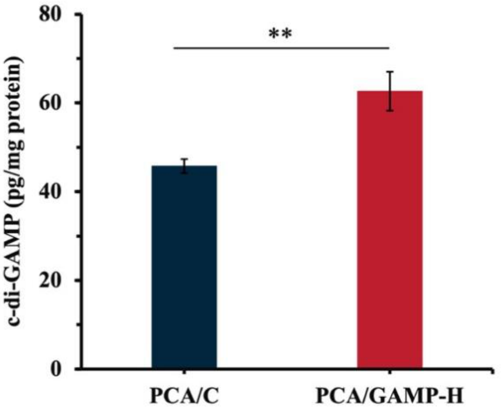

Fig. S3 The intracellular cGAMP concentration in the cells of PCA/C and PCA/GAMP-H. A two-sided Student's *t* test was used to analyze the statistical significance (**p < 0.01).

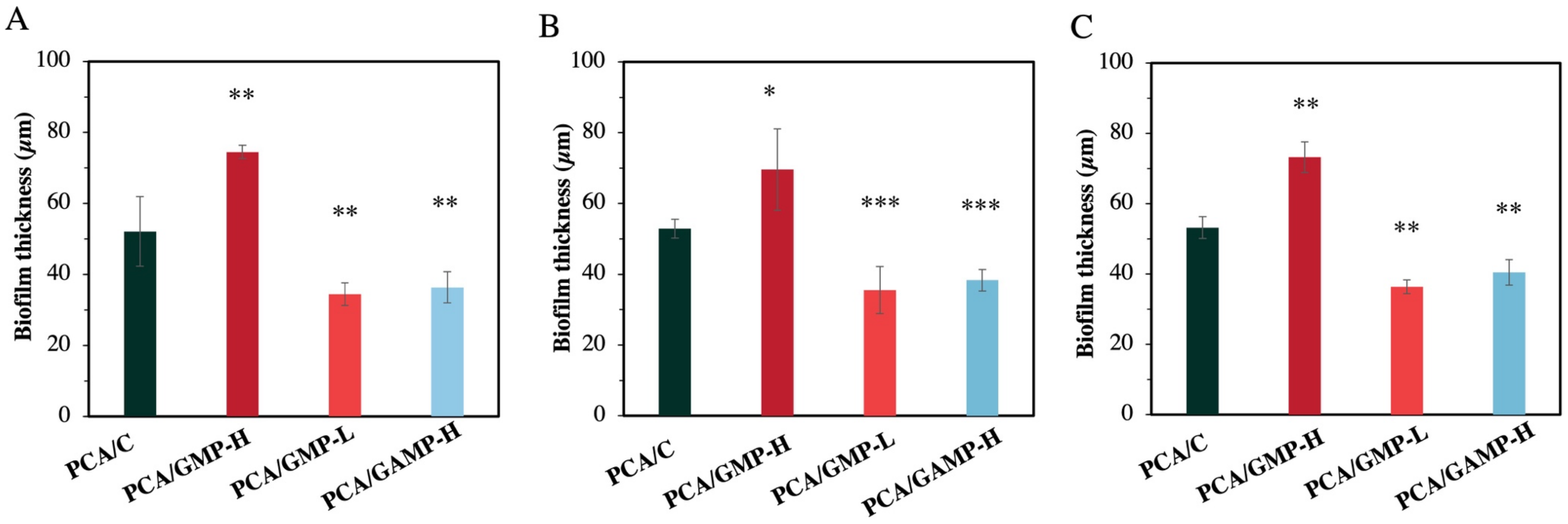

Fig. S4 The average thickness of (A) 6-day-old biofilms on ferrihydrite, (B) 8-day-old biofilms on lepidocrocite, and (C) 6-day-old biofilms on goethite. A two-sided Student's *t* test was used to analyze the statistical significance (*$p < 0.05$, **$p < 0.01$ and ***$p < 0.001$).

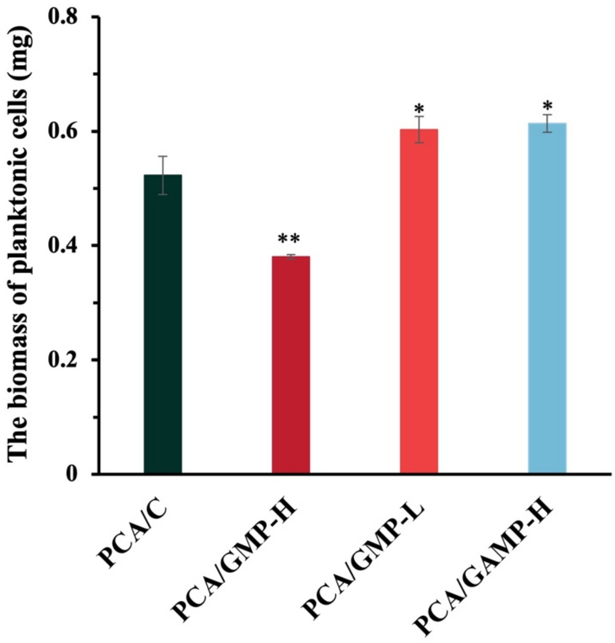

Fig. S5 The biomass of *G. sulfurreducens* planktonic cells involved in biofilm-mediated ferrihydrite reduction was assessed by quantifying the total protein content in the supernatants after a 6-day incubation period. A two-sided Student's *t*-test was employed to evaluate statistical significance, with results denoted as $*p < 0.05$ and $**p < 0.01$.

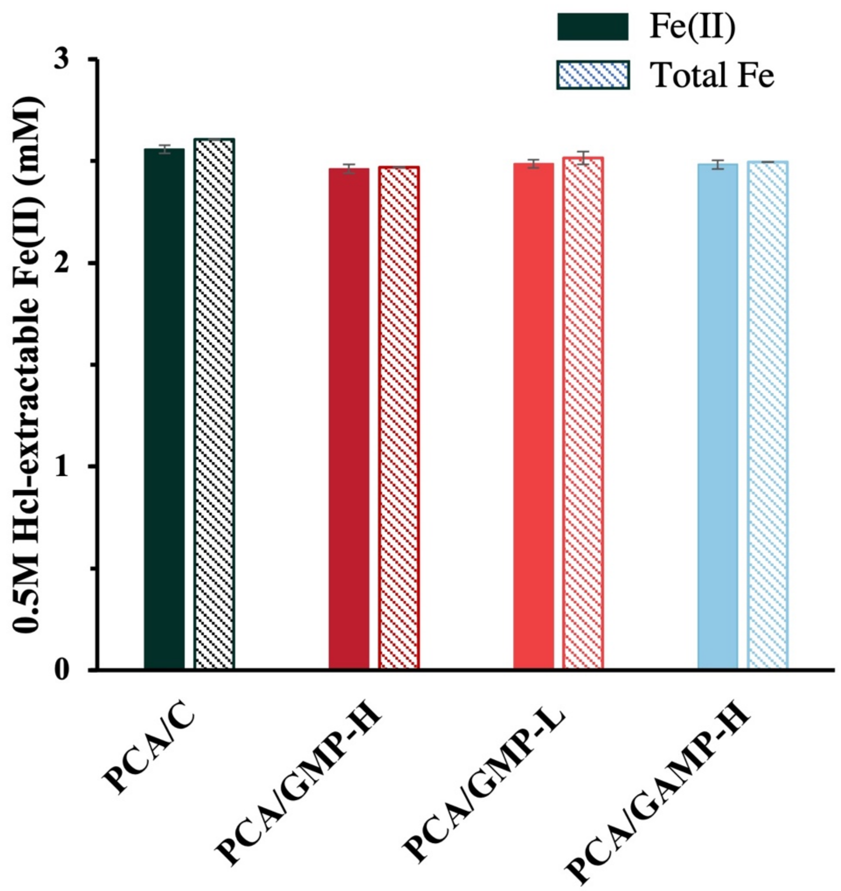

Fig. S6 The concentrations of Fe(II) and total iron in the supernatants of the biofilm-mediated ferrihydrite reduction system after a 6-day incubation period.

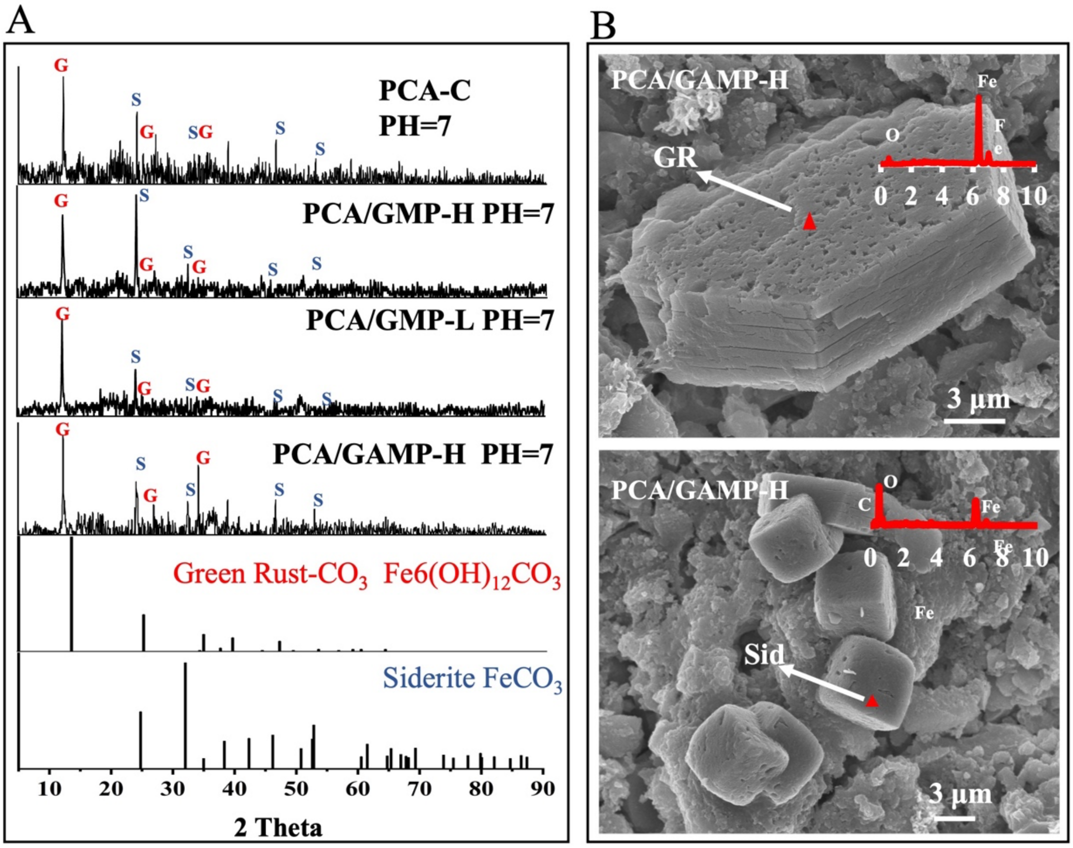

Fig. S7 (A) XRD and (B) SEM of secondary minerals in ferrihydrite-reducing cultures.

Abbreviations: GR (green rust), Sid (siderite).

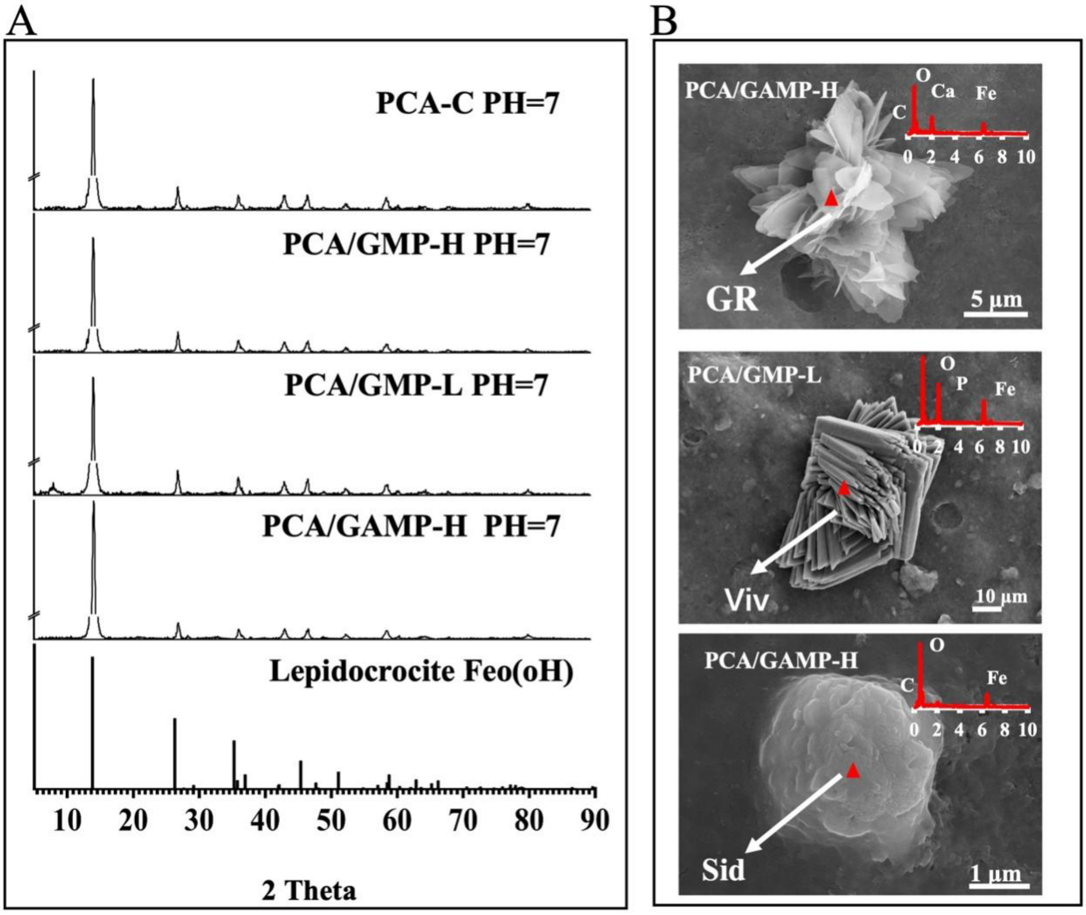

Fig. S8 (A) XRD and (B) SEM of secondary minerals in lepidocrocite-reducing cultures. Abbreviations: GR (green rust), Viv (vivianite), Sid (siderite).

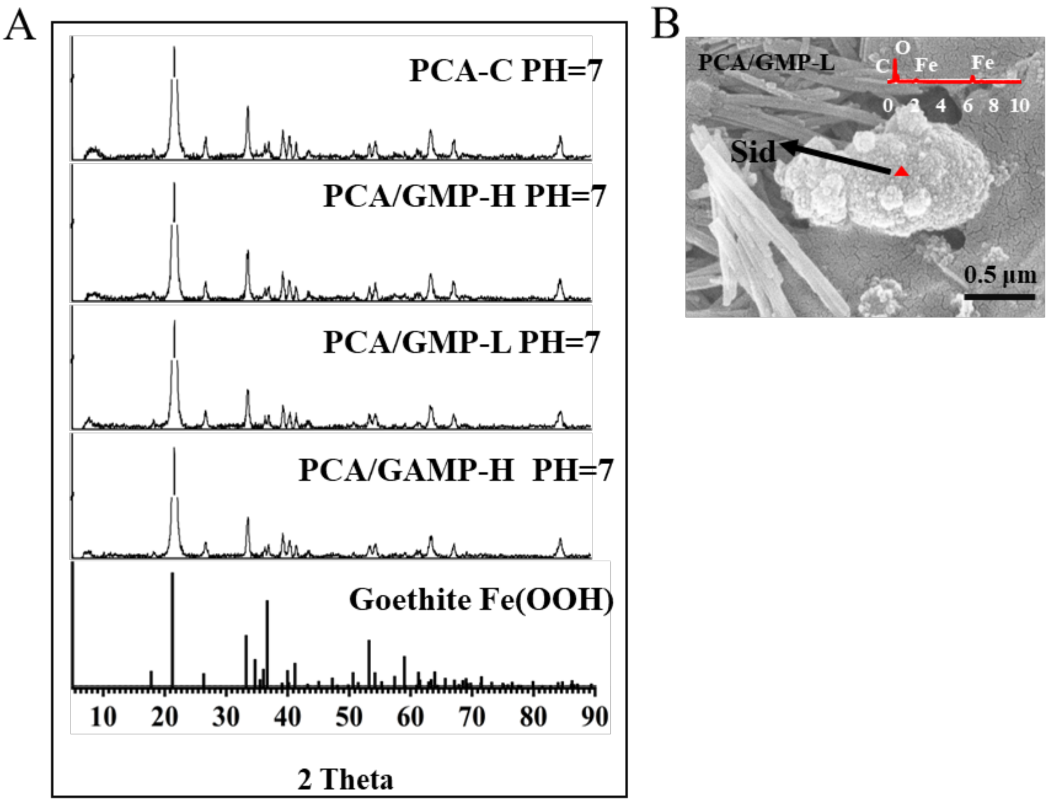

Fig. S9 (A) XRD and (B) SEM of secondary minerals in goethite-reducing cultures. Abbreviations: Sid (siderite).

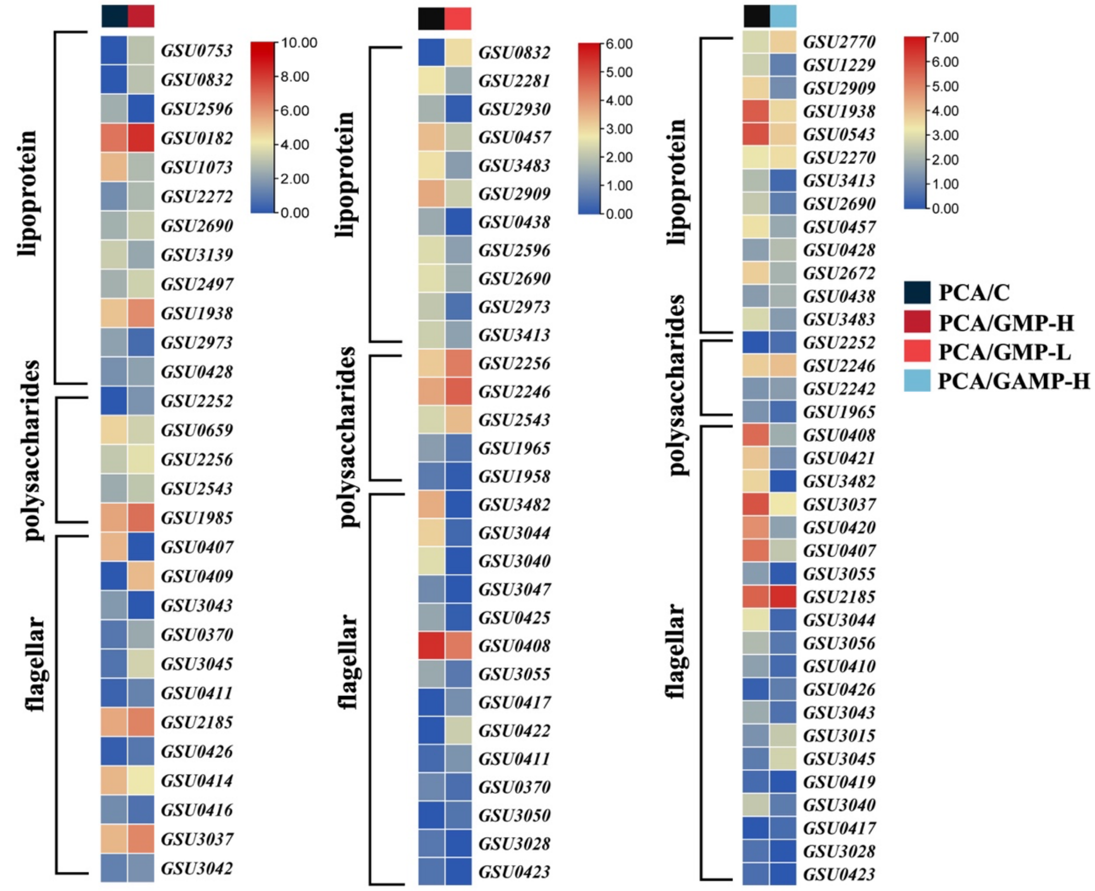

Fig. S10 Expression of the genes associated with lipoproteins, polysaccharide biosynthesis, and flagella.

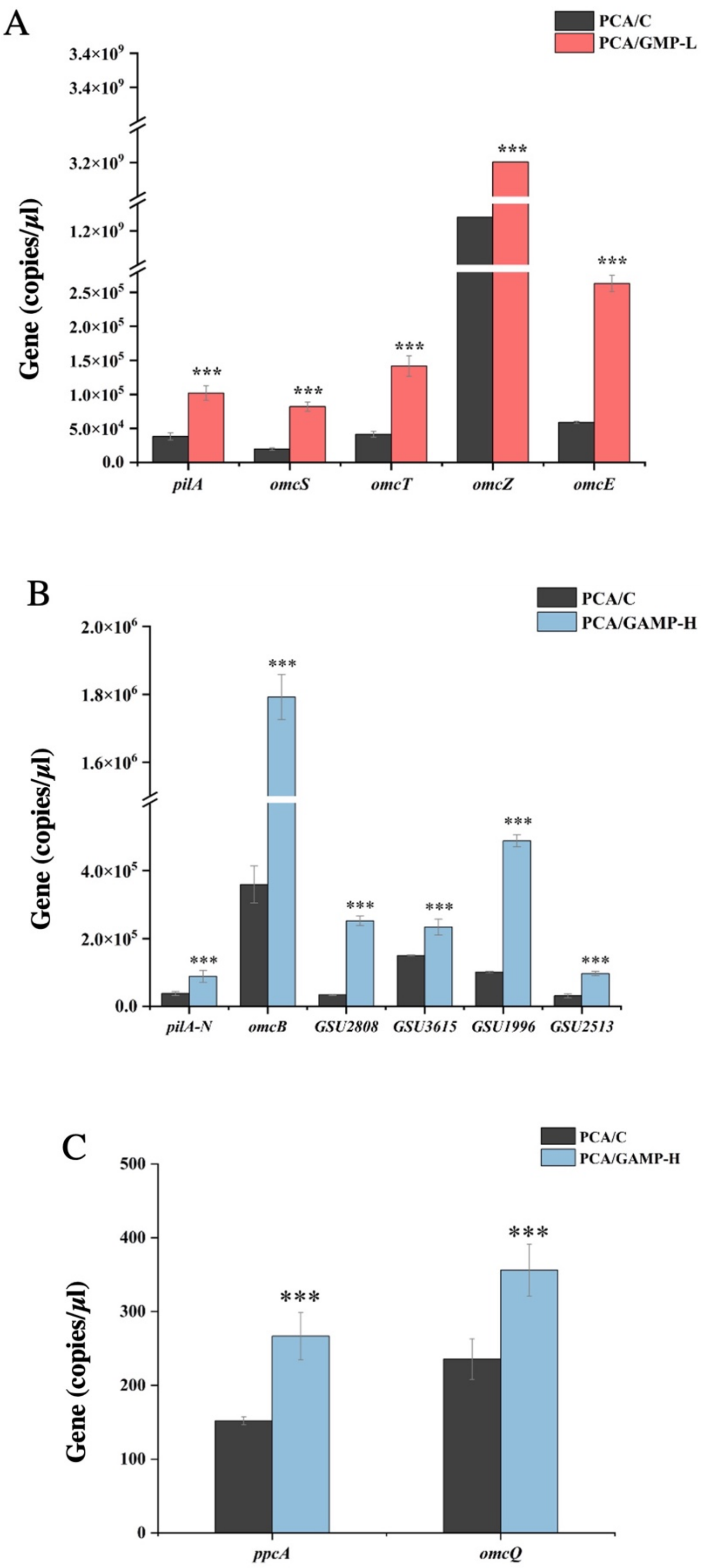

Fig. S11 The qPCR analysis of the expression of genes encoding EET proteins in PCA/GMP-L and PCA/GAMP-H cells. A two-sided Student's t test was used to analyze the statistical significance (**$p < 0.01$, *** $p < 0.001$).

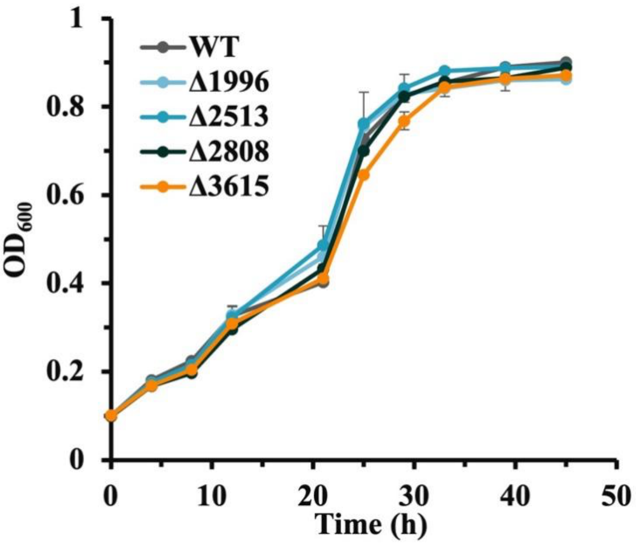

Fig. S12 Cell growth of Δ1996, Δ2513, Δ2808 and Δ3615 and WT *G. sulfurreducens* (n = 3 independent samples) in the medium using fumarate as electron acceptors. Data are shown as the mean ± SD.

Table S1. Strains and plasmids used in this study.

**Strain or plasmid**	**Relevant characteristics**	**Source**
Strain		
G. sulfurreducen PCA	Wild type (WT)	Lab stock
PCA/C	WT carrying the empty vector pYYDT	(1)
PCA/GMP-L	WT carrying pYhjH that overexpresses a c-di-GMP hydrolase	(1)
PCA/GMP-H	WT carrying pYedQ that overexpresses a c-di-GMP synthase	(1)
PCA/GAMP-H	WT carrying pGacA that overexpresses a c-di-GAMP synthase	This study
Δ1996	Deletion of *gsu1996* gene from WT *G. sulfurreducen* genome	This study
Δ2513	Deletion of *gsu2513* gene from WT *G. sulfurreducen* genome	This study
Δ3615	Deletion of *gsu3615* gene from WT *G. sulfurreducen* genome	This study
Δ2808	Deletion of *gsu2808* gene from WT *G. sulfurreducen* genome	This study
Plasmids		
pYYDT	Km^R; *oriV*(pBBR1), P*tac*	(2)
pYhjH	Km^R; *oriV*(pBBR1), P*tac*, *yhjH*	(1)
pYedQ	Km^R; *oriV*(pBBR1), P*tac*, *yedQ*	(1)
pGacA	Km^R; *oriV*(pBBR1), P*tac*, *gacA*	This study
pGSU1996	Km^R; *oriV*(pBBR1), P*tac, gsu1996*	This study
pGSU2513	Km^R; *oriV*(pBBR1), P*tac, gsu2513*	This study

Table S2. The primers used in this study.

Primer	Sequence (5' to 3')	Description
YYD-F	GCCTCAGGCATTTGAGAAGCACA	pYYDT sequence
YYD-R2	TTGACGAGTTCTTCTGAGCG	pYYDT sequence
PUC19-F	CAATAACGAGGAGGA	pUC19 sequence
PUC19-R	CCATGGCGGGGAGCG	pUC19 sequence
GacA-F	AGGAATTCGCGGCCGCTTCTAGAAAAAGAGGAG AAAACTAGAATGGAGC	Clone *gacA* gene
GacA-R	ACCTGCAGGCGGCCGCTACTAGTTTACCGGATGG CGGTGGC	Clone *gacA* gene
2513-1-F	CATGGGCTCACTGTCGATCT	*gsu2513* knockout
2513-1-R	GGCGTACCTCCGTTTGGTA	*gsu2513* knockout
2513-2-F	TACCAAACGGAGGTACGCCCAATAACGAGGAGG A	*gsu2513* knockout
2513-2-R	GGCGAATGGGTGACGTAATCGCTCCCCGCCATGG	*gsu2513* knockout
2513-3-F	ATTACGTCACCCATTCGCC	*gsu2513* knockout
1996-1-F	TCTGCCCATGCTGATAACC	*gsu1996* knockout
1996-1-R	GACGCATTTTCATGATAAGCTCC	*gsu1996* knockout
1996-2-F	GGAGCTTATCATGAAAATGCGTCCAATAACGAGG AGGA	*gsu1996* knockout
1996-2-R	CGGCTGCGTTACATGTTGTCGCTCCCCGCCATGG	*gsu1996* knockout
1996-3-F	ACAACATGTAACGCAGCCG	*gsu1996* knockout
1996-3-R	CAGATCGAGAATGGAGTCGG	*gsu1996* knockout
3615-1-F	CATCCCGGCTGTCTCTAGGAGA	*gsu3615* knockout
3615-1-R	AGGTAGTGGAAGACGTGG	*gsu3615* knockout
3615-2-F	CCACGTCTTCCACTACCTCAATAACGAGGAGGA	*gsu3615* knockout
3615-2-R	GATCAATGGAGGAGCCCTCGCTCCCCGCCATGG	*gsu3615* knockout
3615-3-F	AGGGCTCCTCCATTGATC	*gsu3615* knockout
3615-3-R	CTTACCGTGACCGACAACA	*gsu3615* knockout
2808-1-F	CCAGTACCTTGTCGACCATG	*gsu2808* knockout
2808-1-R	GTCTGCGCATATATCCTCCTG	*gsu2808* knockout
2808-2-F	CAGGAGGATATATGCGCAGACCAATAACGAGGAGG A	*gsu2808* knockout
2808-2-R	TCGTCCTTATCAGTCCCGGCGCTCCCCGCCATGG	*gsu2808* knockout
2808-3-F	CCGGGACTGATAAGGACGA	*gsu2808* knockout
2808-3-R	GATGTGGTGCCACTGGAA	*gsu2808* knockout
QpilA-F	ACTGCTCTTGAGTCCGCATT	qPCR quantification
QpilA-R	ATTGACAGTTCCCGCGGT	qPCR quantification

QomcS-F	CTTGTAGGAGTTGTACAGACCGG	qPCR quantification
QomcS-R	CTACAACCGGACGGAAGCTAC	qPCR quantification
QomcT-F	CGAACATGAAGGCATCGCC	qPCR quantification
QomcT-R	ACCTACCGGATCACCTCACTG	qPCR quantification
QomcZ-F	ACAGTGGGCTGATAGGTGGTC	qPCR quantification
QomcZ-R	ATCCAGAAGGACAGCCCCAAT	qPCR quantification
QomcE-F	CAACGCGCAACAGGACATT	qPCR quantification
QomcE-R	CCGGCACGTTATTCATGCTG	qPCR quantification
QppcA-F	ACATCGTCCTCAAGGCCAAG	qPCR quantification
QppcA-R	GAGCCATCTCTTTGCCGAAG	qPCR quantification
QomcB-F	GATACCATCCGCATCGGTG	qPCR quantification
QomcB-R	CGCAGGTGTTAACTCCACGAG	qPCR quantification
QomcQ-F	CAGGCATTGCAGTTGTCATAGAC	qPCR quantification
QomcQ-R	CCTGCCATAATGGCAAGGAG	qPCR quantification
Q2808-F	CACTAACAACGTCAACCGACACC	qPCR quantification
Q2808-R	GGCTACAGGTGCTGACATTGTG	qPCR quantification
Q3615-F	GTGTGACAGTCGCTGCACTTC	qPCR quantification
Q3615-R	GCAAGCCCTTTACCGACAAG	qPCR quantification
Q1996-F	CCACTCGAACCGTGAGATCAC	qPCR quantification
Q1996-R	CATGCCCTTGTGGCACTTG	qPCR quantification
Q2513-F	GTTCTTGTGGCAGTGATGTCCG	qPCR quantification
Q2513-R	GCACCGCTTGTTGAAGATGG	qPCR quantification

Table S3. The *c*-Cyt genes upregulated in PCA/GMP-L and PCA/GAMP-H cells.

**Locus ID**	**Gene annotation**	**Gene name**	**PCA/GMP-L *vs* PCA/C**		**PCA/GAMP-H *vs* PCA/C**		**Cellular location**
			log2 FC	P-*value*	log2 FC	P-*value*	
**Upregulated *c*-type cytochromes in both PCA/GMP-L and PCA/GAMP-H cells**							
GSU1496	Pilin protein	*pilA-N*	6.685	3.42E-05	6.932	2.73E-06	Extracellular
Upregulated c-type cytochromes in PCA/GMP-L cells							
GSU2294	Cytochrome c family protein, 6 heme-binding sites	*omcM*	1.004	9.97E-06	-	-	Outer membrane
GSU0618	Cytochrome c family protein, 4 heme-binding sites	*omcE*	1.001	2.21E-06	-	-	Extracellular
GSU2076	Cytochrome c family protein, 4 heme-binding sites	*omcZ*	1.276	4.75E-02	-	-	Extracellular
GSU2503	Cytochrome c, 6 heme-binding sites	*omcT*	1.760	1.32E-05	-	-	Extracellular
GSU2504	Cytochrome c family protein, 1 heme-binding site	*omcS*	1.975	1.96E-17	-	-	Extracellular
**Upregulated c-type cytochromes in PCA/GAMP-H cells**							
GSU2513	Cytochrome c family protein, 1 heme-binding site	-	-	-	1.935	5.30E-04	Outer membrane
GSU1996	Cytochrome c family protein, 12 heme-binding sites	-	-	-	1.398	7.86E-05	Periplasmic
GSU3615	Cytochrome c, 7 heme-binding sites	-	-	-	1.170	2.36E-03	Extracellular
GSU2808	Cytochrome *c* family protein, 6 heme-binding sites	-	-	-	1.234	5.07E-09	Outer Membrane

GSU0592	Cytochrome c family protein, 12 heme-binding sites	*omcQ*			1.073	4.33E-07	Inner membrane
GSU0105	Cytochrome *c* family protein, 3 heme-binding sites	-			4.012	2.73E-02	Periplasmic
GSU0612	Cytochrome *c*, 3 heme-binding sites	*ppcA*			1.221	4.64E-05	Periplasmic
GSU0364	Cytochrome c7, 3 heme-binding sites	*ppcB*	-	-	1.292	1.22E-06	Periplasmic

Table S4. Summary of ferrihydrite reduction rates and extents of the gene-deletion mutants.

Strain	Rate (k) ($mmol \cdot L^{-1} \cdot day^{-1}$)^{a,b}	Fraction of Fe(III) substrate reduced (%)^{a,c}
PCA-WT	0.842 ± 0.03	33.4 ± 0.71
Δ1996	0.652 ± 0.03**^{d}	30.3 ± 1.57
Δ2513	0.727 ± 0.01*	27.01 ± 0.84
Δ2808	0.821 ± 0.02 (NS)	33.0 ± 1.75
Δ3615	0.826 ± 0.02 (NS)	32.07 ± 1.11

$^{a.}$ The uncertainties indicate standard deviation of triplicate samples;

$^{b.}$ The rate constants of ferrihydrite were calculated using pseudo-zero-order model for the datasets for 5 days;

$^{c.}$ Calculated as the ratio of 0.5 M HCl-extractable Fe(II) to the starting Fe(III) concentration in the reactor;

$^{d.}$ Statistical significance was determined using two-sided Student's t test for comparing gene-deletion mutants with the WT *G. sulfurreducens*. p values are reported using the following symbolic representation: NS (No significance) $p > 0.05$, *$p < 0.05$, **$p < 0.01$.

References:

1. Hu Y, Han X, Luo Y, Jiang J, Jiang Y, Cao B, Dong Y, Shi L. 2024. All roads lead to Rome: Cyclic di-GMP differentially regulates extracellular electron transfer in *Geobacter* biofilms. The Innovation Life 2:100052.
2. Yang Y, Ding Y, Hu Y, Cao B, Rice SA, Kjelleberg S, Song H. 2015. Enhancing Bidirectional Electron Transfer of *Shewanella oneidensis* by a Synthetic Flavin Pathway. ACS Synthetic Biology 4:815-823.
